# STING agonist-conjugated metal-organic framework induces artificial leukocytoid structures and immune hotspots for systemic antitumor responses

**DOI:** 10.1093/nsr/nwae167

**Published:** 2024-05-10

**Authors:** Taokun Luo, Xiaomin Jiang, Yingjie Fan, Eric Yuan, Jinhong Li, Langston Tillman, Wenbin Lin

**Affiliations:** Department of Chemistry, University of Chicago, Chicago 60637, USA; Department of Chemistry, University of Chicago, Chicago 60637, USA; Department of Chemistry, University of Chicago, Chicago 60637, USA; Department of Chemistry, University of Chicago, Chicago 60637, USA; Department of Chemistry, University of Chicago, Chicago 60637, USA; Department of Chemistry, University of Chicago, Chicago 60637, USA; Department of Chemistry, University of Chicago, Chicago 60637, USA; Department of Radiation and Cellular Oncology and the Ludwig Center for Metastasis Research, University of Chicago, Chicago 60637, USA

**Keywords:** metal-organic framework, radiosensitization, STING agonist, artificial leukocytoid structures, immune hotspots, immunotherapy, nanomedicine

## Abstract

Radiotherapy is widely used for cancer treatment, but its clinical utility is limited by radioresistance and its inability to target metastases. Nanoscale metal-organic frameworks (MOFs) have shown promise as high-Z nanoradiosensitizers to enhance radiotherapy and induce immunostimulatory regulation of the tumor microenvironment. We hypothesized that MOFs could deliver small-molecule therapeutics to synergize with radiotherapy for enhanced antitumor efficacy. Herein, we develop a robust nanoradiosensitizer, GA-MOF, by conjugating a STING agonist, 2′,3′-cyclic guanosine monophosphate–adenosine monophosphate (GA), on MOFs for synergistic radiosensitization and STING activation. GA-MOF demonstrated strong anticancer efficacy by forming immune-cell-rich nodules (artificial leukocytoid structures) and transforming them into immunostimulatory hotspots with radiotherapy. Further combination with an immune checkpoint blockade suppressed distant tumors through systemic immune activation. Our work not only demonstrates the potent radiosensitization of GA-MOF, but also provides detailed mechanisms regarding MOF distribution, immune regulatory pathways and long-term immune effects.

## Teaser

A bifunctional metal-organic framework simultaneously enhances low-dose radiotherapy and delivers innate immunomodulators to induce immune hotspots in tumors, thereby enhancing systemic anti-tumor immunity for tumor regression in murine models.

## INTRODUCTION

Radiotherapy (RT) is one of the most widely used cancer treatments in both curative and palliative settings [1], with approximately half of all cancer patients treated with RT during their disease courses. RT utilizes ionizing radiation to generate reactive radicals and damage DNA, thereby killing fast-proliferating cancer cells [2]. While RT successfully eliminates cancerous cells, it is also highly toxic to normal tissues. Thus, RT is limited by cumulative radiation dose and localized radiation to avoid excessive damage to normal tissues. As a result, RT is mostly applied in a local-regional setting and cannot target distant metastases [3]. Furthermore, recurrent and metastatic tumors can develop radioresistant phenotypes, making RT much less effective for these tumors [4,5].

To improve its efficacy, RT has traditionally been combined with chemotherapy. However, the combination of RT and chemotherapy is also highly toxic to normal tissues, causing debilitating side effects to patients. In the past three decades, significant efforts have been devoted to the search for non-chemotherapy radiosensitizers to augment the efficacy of RT without causing serious adverse effects [6,7]. Of particular interest are high-Z nanoradiosensitizers, which are non-toxic and can enhance RT by absorbing more radiation energy [8,9]. They are administered intratumorally (*i.t.*) to achieve a high local concentration for effective radiosensitization and cancer cell killing [10,11].

Despite preclinical efficacy in enhancing RT-mediated cell killing, non-chemotherapy radiosensitizers have not been approved for clinical use by the US Food and Drug Administration [12]. This lack of clinical success has been attributed to the immunosuppressive properties of RT in the complex tumor microenvironment (TME) [13]. RT often induces an immunosuppressive TME due to increased recruitment of myeloid-derived suppressor cells (MDSCs), upregulation of the nuclear factor kappa-light-chain-enhancer of activated B cells (NF-κβ) and exhaustion of lymphoid cells [14,15]. This immunosuppressive TME is less responsive to RT and prevents the patient's immune system from attacking tumor cells.

Given these limitations of existing radiosensitizers, we have identified nanoscale metal-organic frameworks (MOFs) as efficient and non-toxic nanoradiosensitizers via a unique radiotherapy-radiodynamic therapy (RT-RDT) process [11,16]. The high-Z-metal-based secondary building units (SBUs) in MOFs efficiently absorb X-rays to enhance RT effects (primarily by enhancing hydroxyl radical generation), whereas the photosensitizing ligands in the MOFs are excited for the RDT process (via singlet oxygen generation) [9,17,18]. MOF-mediated RT-RDT causes immunogenic cell death (ICD) of cancer cells to synergize with toll-like receptor agonists and immune checkpoint inhibitors [19–21]. We have also used MOFs for neutrophil reprogramming [21]. As MOFs are known to be a unique class of porous material [22,23], we surmised that MOFs could deliver other small molecule therapeutics to synergize with RT-RDT for enhanced antitumor efficacy. There is also a need to understand the detailed mechanisms of MOF-mediated TME regulation, including MOF distribution in tumors, immune regulation pathways and long-term immune effects after RT-RDT treatment.

Herein, we report a highly effective nanoradiosensitizer, GA-MOF, by conjugating a MOF with an agonist of stimulator of interferon genes (STING), 2′,3′-cyclic guanosine monophosphate–adenosine monophosphate (GA), for synergistic RT and immunotherapy. Coordination between GA and Hf_12_-SBU prolonged GA retention in tumors and enhanced STING activation. GA-MOF showed low preclinical toxicity, and when combined with low-dose RT, demonstrated superb anticancer efficacy in colon cancer, pancreatic cancer, and head and neck cancer models. We determined the uptake of MOF particles by tumor cells and different immune populations, and revealed the formation of immune-cell-rich nodules, termed artificial leukocytoid structures (ALS), in the tumors after *i.t.* injection of GA-MOF. Low-dose RT turned these ALS into immunostimulatory hotspots in the tumors and facilitated antitumor immunity. Further combination of GA-MOF plus RT with an immune checkpoint blockade (ICB) not only improved local cancer eradication, but also suppressed distant tumors via systemic immune activation.

## RESULTS

### MOF enhances RT and enables sustained release of GA

**Figure 1.**
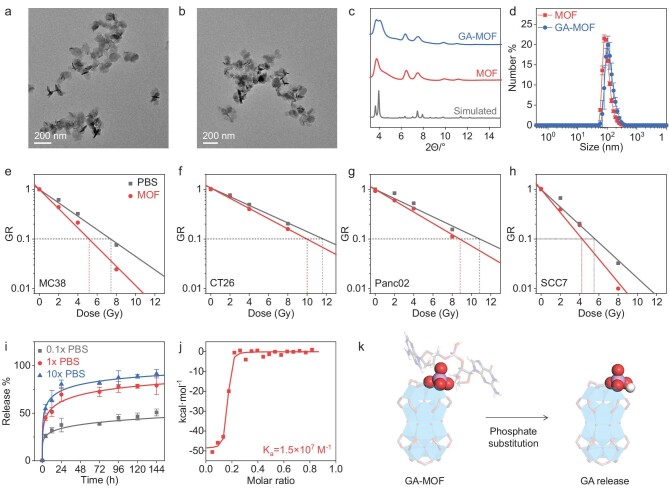
MOF characterization, radiosensitization and GA release profiles. (a and b) TEM images of MOF (a) and GA-MOF (b) (scale bar: 200 nm). (c) PXRD patterns of simulated DBP-Hf, MOF and GA-MOF. (d) Number-average sizes of MOF and GA-MOF by DLS in water. (e–h) GR assays showing radiosensitization effects of MOF in MC38 (e), CT26 (f), Panc02 (g) and SCC7 (h) cell lines. (i) Release percentages of GA from GA-MOF after incubation in 0.1× PBS, 1× PBS and 10× PBS for 6 days. (j) Binding affinity between GA and MOF by ITC analysis. (k) Schematic showing coordination between GA and MOF and the replacement of GA by phosphate anions under physiological conditions. GA (except one phosphate) is shown in a stick model and the phosphate is shown using a space-filling model. Cyan polyhedron, Hf; pink, P; red, O; blue, N; gray, C; white, H.

DBP-Hf MOF was synthesized via a solvothermal reaction between HfCl_4_ and 5,15-di(p-benzoato)porphyrin (H_2_DBP) in *N,N*-dimethylformamide (DMF) with acetic acid (AA) as a modulator (Fig. S1) [22]. The DBP-Hf MOF consisted of Hf_12_ SBUs and photosensitizing DBP ligands for RT-RDT effects [11]. The AA-capped DBP-Hf MOF was modified with trimethylsilyl trifluoroacetate (TMS-TFA) to afford a TFA-capped DBP-Hf MOF (abbreviated as MOF in the following text) (Fig. S1) [19]. This AA/TFA exchange created a dynamic surface for GA coordination [23] to afford GA-MOF by replacing the weakly bound TFA on Hf_12_-SBUs with GA (Figs S2 and S3). Liquid chromatography-mass spectrometry (LC-MS) analysis gave a GA loading of 0.74 wt% in GA-MOF, corresponding to 1 GA molecule per 14.9 Hf_12_-SBUs. Transmission electron microscopy (TEM) revealed the same nanoplate morphology for MOF and GA-MOF (Fig. 1a and b). Powder X-ray diffraction (PXRD) patterns showed that GA-MOF maintained the same crystalline hexagonal close-packed (hcp) topology as MOF (Fig. 1c). Dynamic light scattering (DLS) revealed hydrodynamic sizes of 100.0 ± 2.2 and 133.3 ± 4.5 nm for MOF and GA-MOF, respectively (Fig. 1d). The binding strength between GA and MOF was quantified by isothermal titration calorimetry (ITC), affording an association constant (*K*_GA_) of (1.50 ± 0.79) × 10^7^ M^−1^ (Fig. 1j).

RT enhancement by MOF was evaluated in four different cell lines, including murine colon cancer MC38 and CT26, pancreatic cancer Panc02, and head and neck cancer SCC7, by a growth rate inhibition (GR) assay [24]. The growth inhibition factors (GIFs) were calculated based on real-time growth rates of cancer cells with increasing doses of X-ray (Fig. S4). The cells treated by MOF without RT [denoted MOF(−)] showed minimal growth delay (Fig. 1e and h and Table S1). With X-ray irradiation, MOF treatment [denoted MOF(+)] significantly reduced cell proliferating rates. The GIF values at the 10% GR level were 1.46, 1.17, 1.23 and 1.31 for MC38, CT26, Panc02 and SCC7, respectively. These results suggest that MOF is a biocompatible and efficient nanoradiosensitizer.

We recently showed that high inorganic phosphate concentrations inside cells could trigger the release of coordinated molecules from the SBUs [21,23]. We used LC-MS to determine the release profiles of GA-MOF in 0.1 × phosphate-buffered saline (PBS) (1.18 mM phosphate), 1 × PBS (11.8 mM phosphate) and 10 × PBS (118 mM phosphate) at 37°C to mimic different physiological conditions [25,26]. Incubation of GA-MOF in 0.1 × PBS released <50% of GA in 6 days, while incubation in 1 × PBS and 10 × PBS released 80% and 92% of GA, respectively (Fig. 1i). Since the cytoplasm has ∼10-fold higher free phosphate concentration (∼10 mM) than the interstitial fluid or plasma (∼1 mM) [25,26], GA-MOF slowly releases GA in the interstitial fluid but rapidly releases GA upon endocytosis into cells [27,28]. The release of GA was found to be pH-independent (Fig. S1). These findings suggest that GA can be released from Hf_12_-SBUs by the intracellular/extracellular phosphate gradient (Fig. 1k) [21,23].

### GA-MOF delivers GA and elicits robust STING activation

**Figure 2.**
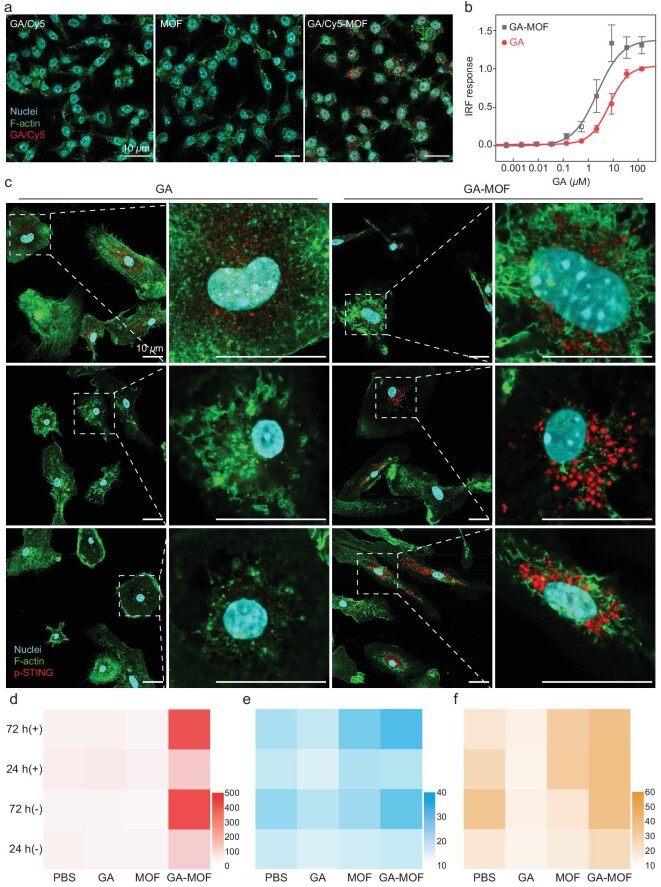
MOF delivers GA for STING activation *in vitro*. (a) Representative CLSM images showing cellular uptake of GA/Cy5 (red) in Raw264.7 cells 24 hours post incubation. (b) EC_50_ of STING activation by GA or GA-MOF. The STING activation was evaluated by its downstream IRF responses with QUANTI-Luc assay in THP-1 reporter cells (*N *= 3). (c) Representative CLSM images showing phosphorylation of STING (p-STING, red) in BMDCs after incubation with GA or GA-MOF for 2, 4 and 8 hours (from top to bottom). For all CLSM images in (a) and (c), the nucleus was stained by Hoechst 33342 in cyan, and the cell morphology was stained by phalloidin in green. Scale bar: 10 *μ*m. (d–f) Heat maps showing secretion levels of IFN-β (d), IL-6 (e) and TNF-α (f) by BMDCs after different treatments (*N *= 3). In each figure, the concentration of the cytokine was given as pg/mL. (+) denotes 2 Gy RT 4 hours post incubation. (−) denotes no RT treatment. The *x*-axis showed treatment groups, and the *y*-axis showed incubation time and whether 2 Gy RT was given (+) or not (−).

The binding of GA to MOF did not affect the cellular uptake of MOF. MOF and GA-MOF showed similar Hf uptake in MC38, CT26 and SCC7 cells (Fig. S5) by inductively coupled plasma-mass spectrometry (ICP-MS). However, GA-MOF significantly enhanced GA uptake *in vitro*. We used cyanine5-conjugated GA (GA/Cy5) to track GA uptake by murine macrophage Raw264.7 cells by confocal laser scanning microscopy (CLSM). GA/Cy5 showed rapid internalization of fluorescence signal in 2 hours, but the signal gradually decreased and almost completely disappeared in 24 hours (Fig. S5). In contrast, the intracellular fluorescence of GA/Cy5-MOF gradually increased and was 34.5-fold stronger than that of the GA/Cy5 group at 24 hours (Fig. 2a). The inhibition experiment showed that only chlorpromazine (but not rottlerin and nystatin) reduced the GA-MOF uptake of Raw264.7 cells, which suggests that Raw264.7 cells take up GA-MOF mainly through clathrin-mediated endocytosis (Fig. S5).

STING activation was assessed using human monocyte THP-1 cells with a luciferase reporter gene linked to interferon (IFN)-responsive elements [29]. After 24-hour incubation, GA-MOF showed a half-maximal effective concentration (EC_50_) for the IFN regulatory factor (IRF) response of 2.34 ± 1.44 *μ*M, which was 3-fold lower than that of GA (EC_50_ = 6.98 ± 1.15 *μ*M) (Fig. 2b). This result indicates that GA-MOF elicits stronger STING activation than GA.

STING activation was supported by phosphorylation of STING (p-STING) by CLSM. Bone-marrow-derived dendritic cells (BMDCs) and bone-marrow-derived macrophages (BMDMs) from C57BL/6 mice were incubated with GA or GA-MOF (0.27* μ*M GA/50 *μ*M Hf) for different lengths of time. GA treatment showed a peak signal of p-STING in BMDCs and BMDMs in 2 hours, while GA-MOF treatment showed sustained STING phosphorylation for up to 8 hours (Fig. 2c and Fig. S6). GA-MOF also showed stronger activation of IRF-3 (Fig. S6) and improved phagocytosis capacity of innate immune cells (Fig. S7).

STING activation leads to the downstream secretion of type-I IFN and proinflammatory cytokines [30]. GA-MOF treatment increased IFN-β secretion in BMDCs, BMDMs and Raw264.7 cells over other groups, indicating stronger activation of the STING-IFN axis in immune cells (Fig. 2d, Fig. S8). Low-dose RT at 2 Gy did not affect IFN secretion, and immune cells were viable upon MOF(+) treatment (Fig. S9). Both GA-MOF(+)-and GA-MOF(−)-treated primary immune cells showed an increased secretion of interleukin-6 (IL-6) and tumor necrosis factor (TNF-α) (Fig. 2e, f and Fig. S8). GA-MOF(+) stimulated the secretion of IFN-β and TNF-α by Raw264.7 cells, but did not upregulate the secretion of immunosuppressive IL-10 and transforming growth factor beta (TGF-β) (Fig. S8). These results demonstrate that GA-MOF induces a more potent and sustained STING activation than GA, leading to enhanced secretion of immunostimulatory cytokines either with or without RT.

### GA-MOF retains GA in tumors and induces artificial leukocytoid structures

**Figure 3.**
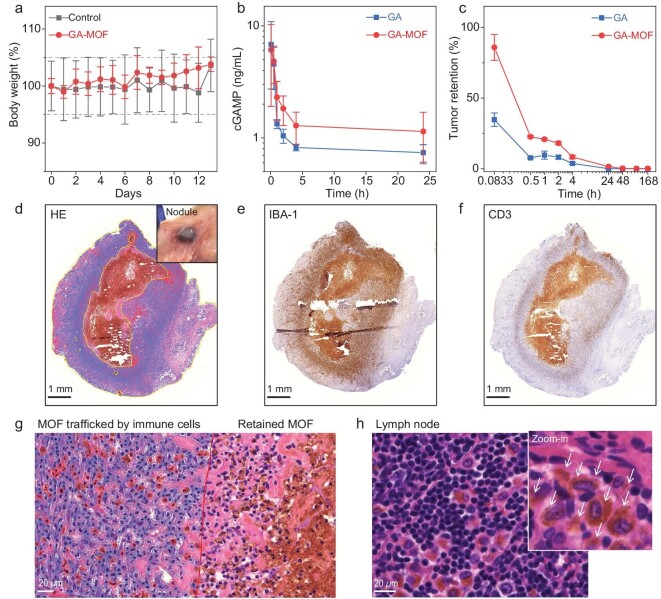
GA-MOF prolongs GA retention and forms immune-cell-rich nodules. (a) Body weight curves of female SD rats after subcutaneous injection of PBS or GA-MOF at a GA dose of 1 mg/kg (*N *= 3). The starting body weight of the rats was ∼200 g. On day 7, the rats received a second subcutaneous injection on the opposite side. (b) Plasma GA concentrations of SD rats after subcutaneous injection of GA or GA-MOF at a GA dose of 1 mg/kg (*N *= 3). (c) Tumor retention percentages of GA after *i.t.* injection of GA or GA-MOF to MC38 tumor-bearing C57BL/6 mice at a GA dose of 1 mg/kg (*N *= 3). (d–f) Representative histological slides of the subcutaneous nodules excised from rats 14 days after injection. The slides were stained with HE (d), IBA-1 (e) and CD3 (f) for cell morphology, innate immune cells and T cells, respectively. Scale bars: 1 mm. In the HE-stained slides (d), the central part with dark brown colors circled by the yellow line corresponds to the retained MOF. The zoomed-in view in (d) is a photo of the subcutaneous nodule. (g) Enlarged HE staining slides of the interface between the MOF part (right) and the cellular part (left) in the nodule. The red line shows an approximate border. Cells carrying MOF (MOF^+^) are marked with red circles. Cells without MOF (MOF^−^) are marked with blue circles. Scale bar: 20 *μ*m. (h) HE staining for the draining LN with a zoomed-in view near the lymphatic vessel (inset, MOF contents are marked by white arrows). Macrophages/monocytes carried GA-MOF back into draining LNs for clearance. Scale bar: 20 *μ*m.

To assess the potential of GA-MOF as an immune agonist and a radiosensitizer, we evaluated the toxicity, pharmacokinetics (PK) and tumor retention of GA-MOF in preclinical models. We injected PBS, GA or GA-MOF at a GA or MOF dose of 1 or 100 mg/kg into the subcutaneous space of Sprague Dawley (SD) rats. The SD rats in all groups showed steady weights and had no health problems throughout the experiment (Fig. 3a and Fig. S10). Compared to GA, GA-MOF showed longer plasma retention of GA, as quantified by a competitive enzyme-linked immunosorbent assay (ELISA) kit (Fig. 3b). The area under the curve (AUC) of GA-MOF in plasma was 1.48-fold higher than that of GA. We quantified the tumor retention of GA after *i.t.* injection of GA or GA-MOF into subcutaneous MC38 tumors in C57BL/6 mice. GA-MOF slowed the release of GA and retained 3-fold more GA in the tumors over a 7-day period (Fig. 3c). These results demonstrate increased GA retention in tumors by GA-MOF.

During preclinical evaluations of SD rats with subcutaneously injected GA-MOF, we observed the formation of dark subcutaneous nodules covered with fascias (Fig. 3d and Fig. S10). We observed similar nodules in MC38 tumors in C57BL/6 mice with *i.t.* injected GA-MOF. To characterize these nodules, we first performed histological staining of the subcutaneous nodules in rats. Due to the dark red color of DBP, we could directly observe MOF by optical microscopy. Hematoxylin and eosin (HE) staining showed the presence of MOF (brown) in the centers and cells and connective tissues in the surrounding areas of the nodules (Fig. 3d). Ionized calcium-binding adapter molecule 1 (IBA-1) and cluster of differentiation 3 (CD3) immunohistochemistry (IHC) staining revealed that the majority of the cells in the nodules were innate immune cells, with some T cells at the nodule margins (Fig. 3e and f and Fig. S11). We hypothesized that GA-MOF acted as a foreign object to attract immune cells into the injection site and activated the local immune system through STING activation [31–33]. Interestingly, we also found significantly less MOF and more cells in the nodules on day 14 (Fig. S11) than on day 7 (Fig. 3d), which suggests active and dynamic transport/clearance of MOF particles by immune cells.

In the HE-stained slides of nodules, ∼16.7% of cells in the nodules carried MOF. Most of these cells were macrophages or monocytes according to their morphologies (Fig. 3e). In the HE-stained slides of draining lymph nodes (LNs), we observed migrating macrophages/monocytes carrying MOF contents (Fig. 3f, Fig. S11). We can depict the fate of GA-MOF after injection into subcutaneous space or tumors based on these observations. After injection, GA-MOF induces immune cell infiltration and triggers foreign body clearance mechanisms. Innate immune cells, including neutrophils, macrophages and monocytes, form the nodules (ALS) surrounding the injected GA-MOF. The phagocytes carry GA-MOF back into the draining LNs for clearance and antigen presentation [34]. T cells are also known to be involved in this clearance and regenerative process [35]. GA may be released once GA-MOF is uptaken by these innate immune cells to activate their STING-related pathways, either in the nodules or in the draining LNs. However, it remains unclear if STING activation contributes to nodule formation.

### RT transforms artificial leukocytoid structures into immune hotspots

**Figure 4.**
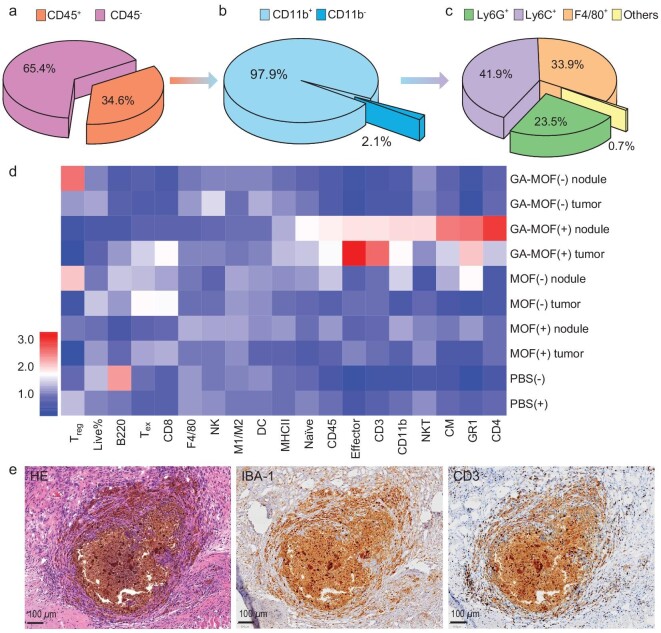
GA-MOF forms ALS and induces immune hotspots with RT. (a–c) Percentages of CD45^+^ or CD45^−^ populations in MOF^+^ cells (a), percentages of CD11b^+^ or CD11b^−^ populations in MOF^+^CD45^+^ cells (b) and percentages of Ly6G^+^ or Ly6C^+^ or F4/80^+^ populations in MOF^+^CD45^+^CD11b^+^ cells (c) (*N *= 5). (d) Heat maps showing immune profiles of tumors and nodules in different treatment groups quantified by flow cytometry (*N *= 3). T_reg_, regulatory T cells. Live%, percentages of live cells in total cells. B220, B cells. T_ex_, exhausted T cells. CD8, cytotoxic T cells. F4/80, macrophages. NK, natural killer cells. M1/M2, the ratio of M1 macrophages to M2 macrophages. DC, dendritic cells. MHCII, MHCII^+^ myeloid cells. Naïve, naïve T cells. CD45, leukocytes. Effector, T effector cells. CD3, T cells. CD11b, myeloid cells. NKT, natural killer T cells. CM, central memory T cells. GR1, granulocytes. CD4, helper T cells. The color scheme is displayed in a linear scale. (e) Histological slides of the intratumoral nodule induced by GA-MOF(+) excised from tumor-bearing mice 14 days after *i.t.* injection. The slides were stained with HE (left, morphology), IBA-1 (middle, phagocytes) and CD3 (right, T cells). Scale bars: 0.1 mm.

Different from the subcutaneous space in rats, MOF after *i.t.* injection can access both tumor cells and immune cells. We performed flow cytometry staining to determine MOF uptake by different cell populations in MC38 tumor-bearing C57BL/6 mice. The fluorescence of DBP (BV711 channel) from MOF was used to differentiate MOF^−^ and MOF^+^ cells in the tumor. One day after *i.t.* injection of MOF, 65.4% MOF^+^ cells were non-immune cells (CD45^−^) and 34.6% MOF^+^ cells were leukocytes (CD45^+^, Fig. 4a). The leukocytes exhibited 1.4-fold higher MOF signal than non-immune cells (Fig. S12). Approximately 97.9% MOF^+^ leukocytes were myeloid cells (MOF^+^CD45^+^CD11b^+^, Fig. 4b), with 41.9% monocytes (Ly6C^+^), 33.9% macrophages (F4/80^+^) and 23.5% neutrophils (Ly6G^+^) (Fig. 4c). These results are consistent with the histological observations of subcutaneous nodules in rats (Fig. 3d and h).

To understand the impact of RT on the nodules, we irradiated the injected tumors with 4 Gy X-ray for 3 daily fractions (Fig. S13), excised the nodules from the tumors 14 days from the first RT, and performed flow cytometric staining. Both MOF and GA-MOF triggered nodule formation in the injected tumors (Fig. S14). These nodules were immunologically distinct from the tumors (Fig. 4d). Compared to GA-MOF(−), MOF(−) and MOF(+), GA-MOF(+) resulted in smaller tumor sizes and a more inflammatory TME with significantly more infiltration of myeloid cells (CD45^+^CD11b^+^), helper T cells (CD45^+^CD3^+^CD4^+^), effector T cells (CD45^+^CD3^+^CD8^+^CD44^+^CD62L^−^) and central memory T cells (CD45^+^CD3^+^CD8^+^CD44^+^CD62L^+^) and downregulation of regulatory T cells (T_reg_, CD45^+^CD3^+^CD4^+^FOXP3^+^CD25^+^) (Fig. 4d, Fig. S14). These results suggest that GA-MOF(+) produces immunologically ‘hot’ nodules (immune hotspots) to suppress tumor growth.

IHC staining of GA-MOF(+)-treated tumors revealed similar nodule structures to the subcutaneous nodules in rats (Figs 3d–f and 4e). The MOF center was surrounded by IBA-1^+^ innate immune cells and CD3^+^ T cells. Colonies of tumor cells were surrounded by a large number of immune cells (Fig. S15), indicating an active and T-cell-dependent antitumor response. In contrast, MOF(+)-treated tumors did not exhibit immune infiltration near the nodules, with isolated MOF islands trapped in the tumor interstices (Figs S14–S16). We also inoculated MC38 tumors in STING-knockout (STING^−/−^) B6 mice, and *i.t.* or subcutaneously injected GA-MOF to observe nodule formation. Compared to wild-type (WT) mice, STING^−/−^ mice showed little infiltration of lymphoid cells in both intratumoral and subcutaneous nodules (Fig. S17). This result indicates that STING activation is required for the formation of immune hotspots in the tumors.

### GA-MOF elicits antitumor effects and activates the TME

**Figure 5.**
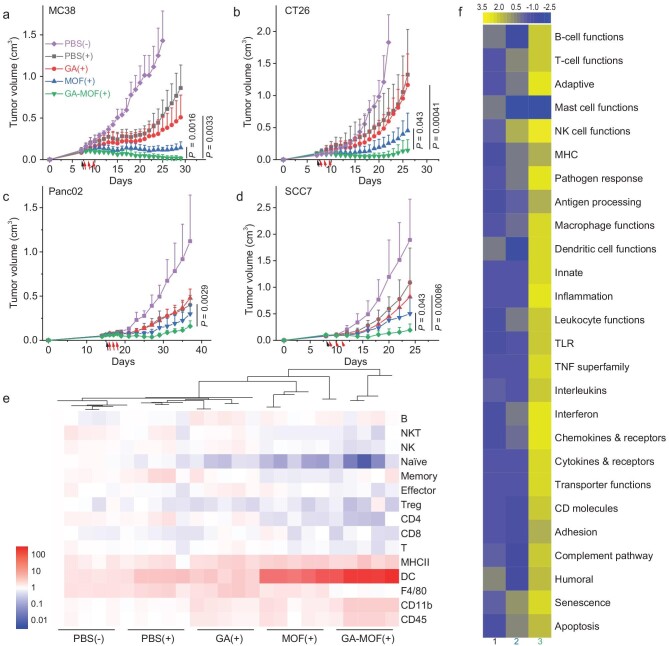
GA-MOF(+) inhibits tumor growth and ameliorates the TME. (a–d) Growth curves of subcutaneous MC38 (a), CT26 (b), Panc02 (c) and SCC7 (d) tumors in different treatment groups (ANOVA with Tukey test, *N *= 7 for MC38, *N *= 8 for CT26, *N *= 5 for Panc02, *N *= 6 for SCC7). The black arrow indicates *i.t.* injection, and the red arrows indicate X-ray irradiation. (e) Heat maps with dendrograms showing immune cell infiltration in tumors on day 13 (3 days after the last RT) quantified by flow cytometry (*N *= 5). The color scheme shows log_10_ fold change. The hierarchical clustering was done by Origin Pro Software with HeatMapDendrogram plug-in. (f) NanoString GSA heat map of directed global significance scores of 1: GA-MOF(−) vs. PBS(−), 2: MOF(+) vs. PBS(−) and 3: GA-MOF(+) vs. PBS(−) with the nCounter PanCancer Immune Profiling Panel (*N *= 3). The scores quantify the degree to which a group of genes is either upregulated or downregulated in relation to the covariate. They were computed by taking the square root of the average signed squared t-statistic for the genes within the gene set. The t-statistics are obtained from the linear regression used in the analysis of differential expression.

We evaluated the antitumor efficacy of GA-MOF in subcutaneous MC38, CT26, Panc02 and SCC7 models. Since different tumor models had different sensitivity to RT, we used 2 Gy by 3 fractions for CT26, 3 Gy by 3 fractions for Panc02 and SCC7, and 4 Gy by 3 fractions for MC38. GA(+) moderately slowed tumor growth with tumor growth inhibition (TGI) values of 53%–64% in the four tumor models. MOF(+) significantly slowed tumor growth with TGI values of 71%–90%. The simple combination of GA and MOF(+) gave a TGI of 79% in the MC38 model (Fig. S18). In stark contrast, GA-MOF(+) treatment synergized RT-RDT effects and STING activation to greatly enhance antitumor efficacy with TGI values of 82%–98% (Fig. 5a–d, Table S2). One out of eight CT26 tumor-bearing mice and five out of seven MC38 tumor-bearing mice were cured after GA-MOF(+) treatment. GA-MOF(−) barely inhibited tumor growth (Fig. S18). Superb antitumor efficacy and steady body weights (Fig. S18) of GA-MOF(+)-treated mice demonstrated GA-MOF as a biocompatible nanoradiosensitizer for synergistic radiosensitization and STING activation. To reveal early changes in the TME, we quantified cytokines in tumor lysates and performed immune profiling of MC38 tumors by flow cytometry 3 days post-treatment (day 13). GA-MOF(+) induced significantly more secretion of IFN-β than GA(+) or PBS(+), but downregulated TGF-β in the tumors (Fig. S19). MOF(+)- and GA-MOF(+)-treated tumors showed >9-fold higher infiltration of dendritic cells (DCs) (CD45^+^CD11b^+^CD11c^+^) than other groups (Fig. 5e, Fig. S20), likely resulting from the enhanced DC differentiation from monocytes and upregulation of CD11c in granulocytes by RT-RDT [11,21,36]. Furthermore, GA-MOF(+) induced 3.4-fold higher DC infiltration and 1.5-fold higher total leukocyte infiltration than MOF(+). The upregulation of major histocompatibility complex class II (MHCII) and CD45, together with the downregulation of naïve T cell marker in the GA-MOF(+) group (Fig. 5e) indicated enhanced antigen presentation and T cell maturation.

To identify the major effector immune populations, we performed depletion experiments with neutralizing antibodies. Depletion of B cells or NK cells did not alter the efficacy of the treatment compared to isotype controls (Fig. S18). However, when the T cells of tumor-bearing mice were depleted by the anti-CD3 antibody, the efficacy of GA-MOF was significantly compromised (Fig. S18). Thus, the GA-MOF-mediated antitumor effect is dependent on T cell responses.

To understand the synergistic effects of STING activation and RT-RDT effects on local tumor regression, we performed TME screening by extracting mRNA from SCC7 tumors for NanoString analysis. Gene set analysis (GSA) revealed that GA-MOF(+) showed higher scores for innate and adaptive immune responses than MOF(+) and GA-MOF(−) (Fig. 5f). In particular, GA-MOF(+) upregulated IFN, proinflammatory cytokines, phagocytosis-related transporter functions, antigen processing and presentation, DC and macrophage functions, and T/B cell functions.

Between MOF(+) and GA-MOF(+)-treated tumors, 51 genes passed the threshold of the nCounter PanCancer Immune Profiling Panel with ≥1.5-fold expression changes and adjacent *p* values (*p*_adj_) < 0.05 (Figs S21 and S22). Compared to MOF(+), GA-MOF(+) increased gene expression downstream of IFN, including radical S-adenosyl methionine domain-containing protein 2 (*Rsad2*, 6.09-fold) and IFN-stimulated gene 20 (*Isg20*, 2.47-fold), and enhanced infiltration and migration of DCs (Fig. 5f), as demonstrated by upregulation of tyrosine-protein kinase *Lyn* (1.92-fold), solute carrier family 11-member 1 (*Slc11a1*, 2.91-fold), C-X-C chemokine receptor type 4 (*Cxcr4*, 3.88-fold) and C-C chemokine ligand 5 (*Ccl5*, 1.84-fold) [37–40]. GA-MOF(+) treatment upregulated *Cd14* (3.42-fold), indicating enhanced differentiation of monocytes into DCs [41]. The expressions of cytokine/chemokine-related genes also showed significant changes. GA-MOF(+)-treated tumors upregulated TNF superfamilies, including *Tnfrsf13b, Tnfrsf1b, Tnfsf12* and *Tnfaip3*, by >1.8-fold over the MOF(+) group. Similar upregulation was observed in C-X-C chemokine ligand 1 (*Cxcl1*, 4.13-fold), *Ccl4* (13.06-fold), *Il1b* (6.72-fold), Pro-Platelet basic protein (*Ppbp*, 6.97-fold), chemokine receptor-like 2 (*Ccrl2*, 8.63-fold), *Cxcl2* (12.84-fold) and *Ccl3* (10.61-fold). Inflammasome pathways were also activated with upregulation of *Nlrp3* (4.01-fold), *Il1b*, IL1 receptor type 2 (*Il1r2*, 6.42-fold), IL1 receptor-associated kinase 3 (*Irak3*, 3.87-fold) and nitric oxide synthase 2 (*Nos2*, 4.18-fold) in GA-MOF(+)-treated tumors. These changes in cytokines/chemokines reflected inflammatory responses of macrophages and active recruitment of leukocytes [42]. STING activation could reprogram tumor vasculature [43,44]. Angiogenesis-related genes were activated in GA-MOF(+)-treated tumors, including *Serpinb2* (9.78-fold), thrombomodulin (*Thbd*, 2.81-fold) and kinase insert domain receptor (*Kdr*, 2.90-fold). The complement system is essential for foreign body clearance and the removal of damaged cells in the innate immune system [45]. Upregulation of *Serping1* (2.74-fold) and complement component 3a receptor 1 (*C3ar1*, 2.24-fold) demonstrated dynamic regulation of the complement system, which might be related to cancer cell apoptosis, nodule formation and MOF trafficking.

### GA-MOF plus **α**PD-L1 elicits robust systemic immune responses

GA-MOF(+) treatment induces intratumoral immune hotspots and synergizes STING activation with RT-RDT for regression of local tumors and amelioration of the TME. Activated immune systems and improved antigen presentation provided excellent contexts for combination with ICB [46]. We established two bilateral tumor models to evaluate the antitumor efficacy and abscopal effects of GA-MOF(+) in combination with a monoclonal anti-PD-L1 antibody (αPD-L1). We treated the primary tumors on the right flanks similarly to the single lateral model and left the distant tumors untreated. The tumor-bearing mice received intraperitoneal (*i.p.*) injections of αPD-L1 (Fig. S13).

**Figure 6.**
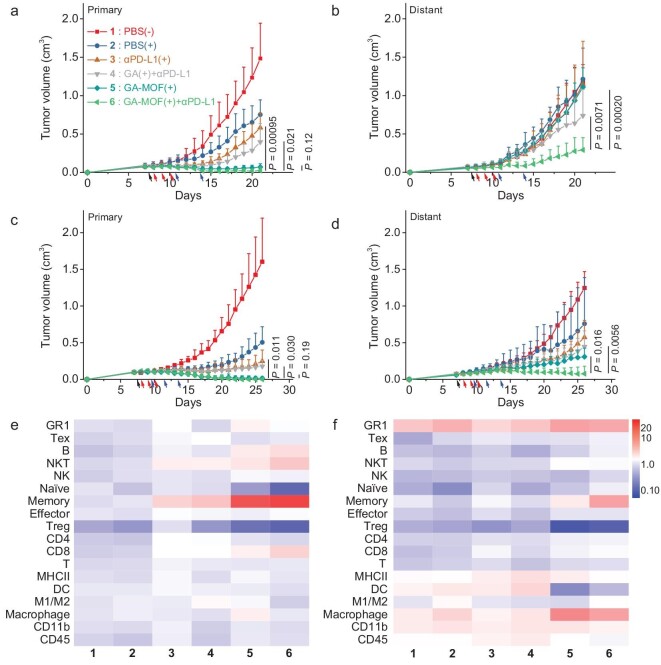
GA-MOF(+) plus αPD-L1 induces systemic antitumor immunity. (a and b) Primary tumor growth curves (a) and distant tumor growth curves (b) of bilateral CT26 tumor-bearing BALB/c mice in different treatment groups (*N *= 6). (c and d) Primary tumor growth curves (c) and distant tumor growth curves (d) of bilateral MC38 tumor-bearing C57BL/6 mice in different treatment groups (*N *= 6). The black arrow indicates particle injection; the red arrows indicate X-ray irradiation; the blue arrows indicate *i.p.* injection of αPD-L1. (e and f) Heat maps showing immune cell infiltration in primary tumors (e) and distant tumors (f) from bilateral MC38 tumor-bearing C57BL/6 mice on day 25, as quantified by flow cytometry (*N *= 6). The color scheme is displayed in a log_10_ scale.

In the bilateral CT26 model, PBS(+) or αPD-L1(+) moderately controlled local tumors with TGI values of <61% and had no effects on distant tumors (Fig. 6a and b, Table S3). GA-MOF(+) exhibited excellent control of primary tumor growth with a TGI of 95% but had no effect on distant tumors. The addition of αPD-L1 to GA-MOF(+) not only enhanced local tumor regression to give a TGI of 98% but also controlled distant tumors with a TGI of 66%. The combination of GA(+) and αPD-L1 also moderately controlled both local and distant tumors with TGI values of 75% and 41%, respectively. This result supports the role of ICB in activating cytotoxic T cells for systemic antitumor effects.

In the bilateral MC38 model, PBS(+) slightly inhibited the growth of primary and distant tumors with TGI values of 66% and 30%, respectively (Fig. 6c and d, Table S3). The αPD-L1(+) treatment enhanced local tumor control with a TGI of 82% and moderately controlled distant tumors with a TGI of 47%. The addition of GA to αPD-L1(+) increased TGI values to 88% and 64% for local and distant tumors, respectively. GA-MOF(+) showed excellent regression of primary tumor growth with a TGI of 99% but had a modest impact on distant tumors with a TGI of 67%. Combination of GA-MOF(+) with αPD-L1 completely eradicated primary (100% TGI) and regressed distant tumors with a TGI of 93% and a 50% cure rate. The combination regimen did not show side effects in both models (Fig. S23).

Flow cytometric profiling of immune cells at the late stage (day 25) showed that GA-MOF(+) enhanced *i.t.* infiltration of adaptive immune cells, particularly memory T cells, CD8^+^ cytotoxic T cells and NKT cells (Fig. 6e, Fig. S24). GA-MOF(+) plus αPD-L1 further enhanced systemic immune responses with increased effector T cells and memory T cells in the distant tumors (Fig. 6f). The reduced T_reg_ populations in the primary and distant tumors of GA-MOF(+) and GA-MOF(+) plus αPD-L1 groups (Fig. 6f) implied a more active T-cell-dependent antitumor effect. Interestingly, the percentages of myeloid cells, including the GR1^+^ population and macrophages, increased in the distant tumors of GA-MOF(+) and GA-MOF(+) plus αPD-L1 groups (Fig. 6f). The enhanced infiltration of innate immune cells is consistent with amelioration of immunosuppressive TMEs and active immune clearance of tumor cells.

**Figure 7.**
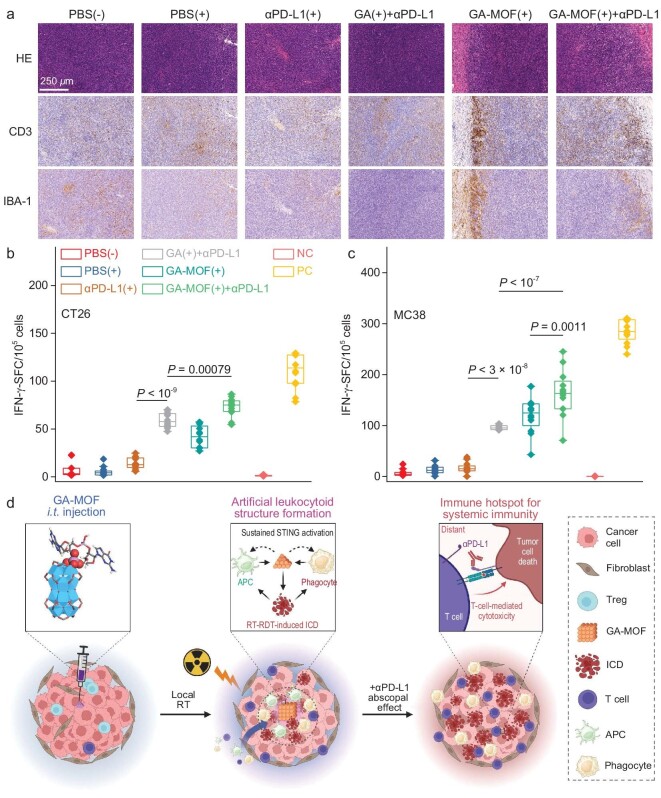
GA-MOF(+) plus αPD-L1 elicits tumor-specific immune responses at distant sites. (a) HE staining (top) of viable cells and CD3 (middle) and IBA-1 (bottom) IHC staining of pan-macrophage cells and T cells, respectively, in distant MC38 tumors. Scale bar: 250 *μ*m. (b and c) ELISpot assays detecting CT26 tumor-specific (b) and MC38 tumor-specific (c) IFN-γ secreting splenocytes. The splenocytes from CT26-bearing BALB/c and MC38-bearing C57BL/6 mice were stimulated with AH1 and KSP, respectively. NC, negative control with no stimulants; PC, positive control stimulated by anti-CD3e and anti-CD28 antibodies; *N *= 6. (d) Proposed mechanism for systemic antitumor responses induced by RT treatment of ALS. GA-MOF attracts infiltration of phagocytes and antigen-presenting cells (APCs). Sustained GA release activates STING in the local TME. MOF-mediated RT-RDT induces ICD of cancer cells to expose antigens for antigen processing and presentation. After GA-MOF(+) treatment, the distant untreated tumor also exhibits active immune infiltration with tumor-specific immune responses. Combination with ICB reinvigorates T cells for adaptive clearance of tumors. (d) was created with BioRender.com.

Analysis of IBA-1 and CD3 markers revealed spatial distributions of innate and adaptive immune cells in the primary and distant tumors. GA-MOF(+) plus αPD-L1 further increased T cell infiltration without impacting the innate immune populations in distant MC38 tumors (Fig. 7a, Fig. S25). This result indicates that GA-MOF(+) plus αPD-L1 reverses immunosuppression to enhance immune cell infiltration and antitumor responses.

We also performed enzyme-linked immunosorbent spot (ELISpot) assays to assess antigen-specific immune responses of T cells (Fig. S26). Splenocytes were isolated from bilateral CT26-bearing BALB/c mice and bilateral MC38-bearing C57BL/6 mice and stimulated with tumor-specific peptide antigens AH1 (SPSYVYHQF) and KSP (KSPWFTTL), respectively. In both models, GA-MOF(+) plus αPD-L1 showed significantly more spot-forming cells (SFCs) than GA-MOF(+) or GA(+) plus αPD-L1, indicating enhanced adaptive immunity to produce more IFN-γ-generating splenocytes and stronger immune memory effects (Fig. 7b and c). The enhanced recognition of tumor antigens correlated well with improved antigen presentation (Fig. 5f) and increased infiltration of memory T cells in the tumors after GA-MOF(+) plus αPD-L1 treatment (Fig. 6e and f). These results show that the synergistic combination induces an inflammatory and T cell infiltrated TME and activates systemic antitumor immune responses for abscopal effects.

## DISCUSSION

Although RT has been in clinical practice for over a century, non-toxic radiosensitizers have yet to be approved by the US Food and Drug Administration for clinical use [47]. Chemotherapeutics such as cisplatin, paclitaxel and gemcitabine are used in combination with RT to enhance antitumor efficacy, but these chemoradiotherapy regimens cause debilitating adverse effects [48,49]. Oxygen mimetics and hypoxia-targeting reagents have been reported to enhance RT in preclinical models, but they failed to demonstrate clinical benefits in human patients [6].

By absorbing more X-rays, high-Z particles increase ionizing events, generation of reactive radicals and DNA damage by RT [50]. This mode of radiosensitization requires very high *i.t.* concentrations of high-Z particles to be effective, which cannot be achieved with existing systemic drug delivery [51–54]. As a result, the nanoradiosensitizers in clinical testing are administered by *i.t.* injection to achieve high local concentrations for cancer killing [55]. However, little is known about the trafficking and clearance of these particles and their induced immune effects [56–60].

As a bifunctional particle, GA-MOF comprises MOF for radiosensitization and the endogenous STING agonist GA for STING activation. The STING pathway elicits antitumor functions such as type-I IFN-mediated T-cell-dependent tumor eradication, modulation of the vasculature and augmentation of adaptive immunity by developing tertiary lymphoid structures [43,61,62]. In this work, we determined the uptake of MOF-based nanoradiosensitizers by different cell populations and their spatial-temporal distributions in tumors. One day post *i.t.* injection, two-thirds of the MOF is uptaken by cancer cells, and the rest of the MOF is uptaken by innate immune cells, including neutrophils, monocytes and macrophages (Fig. 4a–c). MOF-mediated RT-RDT is non-toxic to terminally differentiated immune cells (Fig. S9) but significantly inhibits cancer cell proliferation (Fig. 1e–h). The MOF particle distribution in cancer cells ensures efficient radiosensitization for cancer cell killing, while MOF uptake by immune cells provides an excellent avenue to deliver innate immune agonists. Indeed, GA-MOF prolongs GA retention in tumors to enhance STING activation of innate immune populations and facilitate antitumor immunity. GA-MOF treatment caused little health burden to murine subjects, exemplified by healthy morphology in major organ histology (Fig. S27), normal liver and kidney functions (Fig. S28), and little deposition of MOF particles in peripheral tissues (Fig. S29).

GA-MOF(+) induces intratumoral immune hotspots and efficiently inhibits tumor growth *in vivo*. We first observed nodule formation after subcutaneous injection and *i.t.* injection of GA-MOF to rats and mice, respectively (Fig. 3d). These nodules contained MOF in the center and innate and adaptive immune cells in the periphery, effectively forming a separated immune space from the rest of the tumor (Fig. 4d and e). These ALS are dominated by innate immune cells (Fig. S14). Although both MOF and GA-MOF can trigger nodule formation in the tumors, only GA-MOF(+) results in an immunologically ‘hot’ TME with immunostimulatory signatures (Figs 4d and 5e and f). The immune hotspots in the tumors disrupt the immunosuppressive TME to elicit antitumor immunity. GA-MOF(+) treatment suppresses tumor growth in four tumor models, including immunologically ‘cold’ Panc02 pancreatic cancer and SCC7 head and neck cancer models. Thus, GA-MOF(+) creates immune hotspots to turn ‘cold’ TME ‘hot’ for enhanced anticancer effects.

Figure 7d summarizes our hypothesis for immune hotspot formation and its role in antitumor immunity. As a foreign object, MOF triggers the complement pathway and attracts immune cells. Adsorption of albumin and other interstitial proteins on the MOF surface triggers immune cell recognition and infiltration [56]. Significant upregulation of complement-related genes, chemokines and adhesion molecules causes active and continuous recruitment of leukocytes into MOF-treated tumors (Fig. 5f, Fig. S22), leading to the formation of ALS. Upon low-dose X-rays, MOF(+) induces ICD of cancer cells and generates autologous tumor-specific antigens *in situ* (Fig. S5) [46,63]. The released GA from GA-MOF induces robust and sustained STING activation (Fig. 2b–f) and facilitates DC infiltration (Fig. 5e), antigen processing and antigen presentation (Fig. 5f).

By inducing ALS formation, GA-MOF not only serves as a nanoradiosensitizer to kill cancer cells and expose tumor-specific antigens *in situ*, but also acts as an exogenous nano-adjuvant to induce immune cell infiltration and enhance antigen presentation [64]. STING activation can also induce vascular disruption and reprogram the tumor protective mechanisms that otherwise inhibit immune cell infiltration. Most importantly, sustained STING activation results in STING-IFN-T-cell-dependent responses (Figs 6e and f, and 7b and c). GA-MOF(+) treatment improved T cell function via antigen presentation, as evidenced by enhanced infiltration of memory T cells (Fig. 6e and f) and improved tumor antigen-specific antitumor responses (Fig. 7b and c). The combination of GA-MOF(+) with αPD-L1 extends this local treatment regimen to systemic antitumor effects. We used bilateral tumor models to mimic patients with distant metastases that are not amenable to localized RT treatment. Blockade of the PD-1/PD-L1 axis not only reinvigorates T cells and eradicates local tumors (Fig. 6a and c), but also reduces the infiltration barrier of T cells to distant tumors and elicits robust abscopal effects (Fig. 6b and d).

Our current study has limitations. The subcellular localization of GA and GA-MOF may not be accurate because we are using GA/Cy5 to visualize GA and the particle, which has different hydrophobicity from GA. The interactions between different immune cells, especially early infiltrated phagocytes, DCs and resident T cells in ALS, were not elucidated. STING polarization of innate immune populations and the contribution of vascular reprogramming also merit further study. Detailed genomic analysis will also help to elucidate these immune pathways and interactions. A detailed toxicology study on large animals such as monkeys or dogs is needed to confirm the biocompatibility of MOF-based nanoradiosensitizers.

In summary, we designed a bifunctional nanoplatform for simultaneous STING activation and radiosensitization. GA-MOF prolongs GA retention in tumors and elicits strong and sustained STING activation. GA-MOF forms ALS that are rich in immune cells, which are converted to immune hotspots upon X-ray irradiation. GA-MOF(+) greatly ameliorates the immunosuppressive TME and demonstrates great local tumor control in four tumor models. Further combination with αPD-L1 elicits a robust abscopal effect, extending this local tumor control to systemic antitumor responses. With the ability to induce ALS, MOFs can not only enhance RT but also provide a unique mechanism for immune cell infiltration and *in situ* vaccination. MOFs thus represent a unique nanoplatform for developing innovative cancer treatments.

## MATERIALS AND METHODS

For detailed materials and methods, please see the Supplementary Notes. The study protocol involving animals was reviewed and approved by the Institutional Animal Care and Use Committee at the University of Chicago (ACUP-72408).

## Supplementary Material

nwae167_Supplemental_File

## Notes

***Conflict of interest statement.*** W.L. is the founder of Coordination Pharmaceuticals Inc., which licensed the nMOF-mediated RT-RDT technology from the University of Chicago.

## References

[1] Chandra RA, Keane FK, Voncken FEM et al. Contemporary radiotherapy: present and future. Lancet 2021; 398: 171–84. 10.1016/S0140-6736(21)00233-6 34166607

[2] Huang R-X, Zhou P-K. DNA damage response signaling pathways and targets for radiotherapy sensitization in cancer. Signal Transduct Target Ther 2020; 5: 60. 10.1038/s41392-020-0150-x 32355263 PMC7192953

[3] Formenti SC, Demaria S. Systemic effects of local radiotherapy. Lancet Oncol 2009; 10: 718–26. 10.1016/S1470-2045(09)70082-8 19573801 PMC2782943

[4] Price JM, Prabhakaran A, West CML. Predicting tumour radiosensitivity to deliver precision radiotherapy. Nat Rev Clin Oncol 2023; 20: 83–98. 10.1038/s41571-022-00709-y 36477705

[5] Schaue D, McBride WH. Opportunities and challenges of radiotherapy for treating cancer. Nat Rev Clin Oncol 2015; 12: 527–40. 10.1038/nrclinonc.2015.120 26122185 PMC8396062

[6] Oronsky BT, Knox SJ, Scicinski J. Six degrees of separation: the oxygen effect in the development of radiosensitizers. Transl Oncol 2011; 4: 189–98. 10.1593/tlo.11166 21804913 PMC3140005

[7] Lawrence TS, Blackstock AW, McGinn C. The mechanism of action of radiosensitization of conventional chemotherapeutic agents. Semin Radiat Oncol 2003; 13: 13–21. 10.1053/srao.2003.50002 12520460

[8] Kuncic Z, Lacombe S. Nanoparticle radio-enhancement: principles, progress and application to cancer treatment. Phys Med Biol 2018; 63: 02TR01. 10.1088/1361-6560/aa99ce 29125831

[9] Xu Z, Ni K, Mao J et al. Monte Carlo simulations reveal new design principles for efficient nanoradiosensitizers based on nanoscale metal–organic frameworks. Adv Mater 2021; 33: 2104249. 10.1002/adma.202104249 PMC8492529 34432917

[10] Yom SS, Takacsi-Nagy Z, Liem X et al. NANORAY-312: a phase III pivotal study of NBTXR3 activated by investigator's choice of radiotherapy alone or radiotherapy in combination with cetuximab for platinum-based chemotherapy-ineligible elderly patients with locally advanced HNSCC. Int J Radiat Oncol Biol Phys 2022; 114: e313. 10.1016/j.ijrobp.2022.07.1372

[11] Lu K, He C, Guo N et al. Low-dose X-ray radiotherapy–radiodynamic therapy via nanoscale metal–organic frameworks enhances checkpoint blockade immunotherapy. Nat Biomed Eng 2018; 2: 600–10. 10.1038/s41551-018-0203-4 31015630

[12] Lenders V, Koutsoumpou X, Sargsian A et al. Biomedical nanomaterials for immunological applications: ongoing research and clinical trials. Nanoscale Adv 2020; 2: 5046–89. 10.1039/D0NA00478B 36132021 PMC9418019

[13] Barker HE, Paget JTE, Khan AA et al. The tumour microenvironment after radiotherapy: mechanisms of resistance and recurrence. Nat Rev Cancer 2015; 15: 409–25. 10.1038/nrc3958 26105538 PMC4896389

[14] Bakhoum SF, Ngo B, Laughney AM et al. Chromosomal instability drives metastasis through a cytosolic DNA response. Nature 2018; 553: 467–72. 10.1038/nature25432 29342134 PMC5785464

[15] Liang H, Deng L, Hou Y et al. Host STING-dependent MDSC mobilization drives extrinsic radiation resistance. Nat Commun 2017; 8: 1736. 10.1038/s41467-017-01566-5 29170400 PMC5701019

[16] Koshy M, Spiotto M, Feldman LE et al. A phase 1 dose-escalation study of RiMO-301 with palliative radiation in advanced tumors. J Clin Oncol 2023; 41: 2527. 10.1200/JCO.2023.41.16_suppl.2527

[17] Wang C, Wang W, Tan J et al. Coordination-based molecular nanomaterials for biomedically relevant applications. Coord Chem Rev 2021; 438: 213752. 10.1016/j.ccr.2020.213752

[18] Banerjee S, Lollar CT, Xiao Z et al. Biomedical integration of metal–organic frameworks. Trends Chem 2020; 2: 467–79. 10.1016/j.trechm.2020.01.007

[19] Ni K, Luo T, Culbert A et al. Nanoscale metal–organic framework co-delivers TLR-7 agonists and anti-CD47 antibodies to modulate macrophages and orchestrate cancer immunotherapy. J Am Chem Soc 2020; 142: 12579–84. 10.1021/jacs.0c05039 32658476

[20] Ni K, Lan G, Chan C et al. Nanoscale metal-organic frameworks enhance radiotherapy to potentiate checkpoint blockade immunotherapy. Nat Commun 2018; 9: 2351. 10.1038/s41467-018-04703-w 29907739 PMC6003951

[21] Luo T, Nash GT, Jiang X et al. A 2D nanoradiosensitizer enhances radiotherapy and delivers STING agonists to potentiate cancer immunotherapy. Adv Mater 2022; 34: 2110588. 10.1002/adma.202110588 PMC9529854 35952624

[22] Lu K, He C, Lin W. Nanoscale metal–organic framework for highly effective photodynamic therapy of resistant head and neck cancer. J Am Chem Soc 2014; 136: 16712–15. 10.1021/ja508679h 25407895 PMC4277757

[23] Luo T, Fan Y, Mao J et al. Metal-organic layer delivers 5-aminolevulinic acid and porphyrin for dual-organelle-targeted photodynamic therapy. Angew Chem Int Ed 2023; 62: e202301910. 10.1002/anie.202301910 PMC10325034 36997341

[24] Xu Z, Luo T, Mao J et al. Monte Carlo simulation-guided design of a thorium-based metal–organic framework for efficient radiotherapy-radiodynamic therapy. Angew Chem Int Ed 2022; 61: e202208685. 10.1002/anie.202208685 PMC9647855 36149753

[25] Bowen JW, Levinson C. Phosphate concentration and transport in Ehrlich ascites tumor cells: effect of sodium. J Cell Physiol 1982; 110: 149–54. 10.1002/jcp.1041100207 7068772

[26] Baker SB, Worthley LI. The essentials of calcium, magnesium and phosphate metabolism: part I. Physiology. Crit Care Resusc 2002; 4: 301–6. 16573443

[27] Chazot G, Lemoine S, Kocevar G et al. Intracellular phosphate and ATP depletion measured by magnetic resonance spectroscopy in patients receiving maintenance hemodialysis. J Am Soc Nephrol 2021; 32: 229–37. 10.1681/ASN.2020050716 33093193 PMC7894675

[28] Auesukaree C, Homma T, Tochio H et al. Intracellular phosphate serves as a signal for the regulation of the PHO pathway in saccharomyces cerevisiae. J Biol Chem 2004; 279: 17289–94. 10.1074/jbc.M312202200 14966138

[29] Xu C, Dobson HE, Yu M et al. STING agonist-loaded mesoporous manganese-silica nanoparticles for vaccine applications. J Control Release 2023; 357: 84–93. 10.1016/j.jconrel.2023.03.036 36948420 PMC10164691

[30] Demaria O, Cornen S, Daëron M et al. Harnessing innate immunity in cancer therapy. Nature 2019; 574: 45–56. 10.1038/s41586-019-1593-5 31578484

[31] Veiseh O, Doloff JC, Ma M et al. Size- and shape-dependent foreign body immune response to materials implanted in rodents and non-human primates. Nat Mater 2015; 14: 643–51. 10.1038/nmat4290 25985456 PMC4477281

[32] Anderson JM, Rodriguez A, Chang DT. Foreign body reaction to biomaterials. Semin Immunol 2008; 20: 86–100. 10.1016/j.smim.2007.11.004 18162407 PMC2327202

[33] Dievernich A, Achenbach P, Davies L et al. Characterization of innate and adaptive immune cells involved in the foreign body reaction to polypropylene meshes in the human abdomen. Hernia 2022; 26: 309–23. 10.1007/s10029-021-02396-7 33788008 PMC8881270

[34] von Andrian UH, Mempel TR. Homing and cellular traffic in lymph nodes. Nat Rev Immunol 2003; 3: 867–78. 10.1038/nri1222 14668803

[35] Adusei KM, Ngo TB, Sadtler K. T lymphocytes as critical mediators in tissue regeneration, fibrosis, and the foreign body response. Acta Biomater 2021; 133: 17–33. 10.1016/j.actbio.2021.04.023 33905946

[36] Guo N, Ni K, Luo T et al. Reprogramming of neutrophils as non-canonical antigen presenting cells by radiotherapy–radiodynamic therapy to facilitate immune-mediated tumor regression. ACS Nano 2021; 15: 17515–27. 10.1021/acsnano.1c04363 34709030

[37] Chu C-L, Lowell CA. The Lyn tyrosine kinase differentially regulates dendritic cell generation and maturation1. J Immunol 2005; 175: 2880–9. 10.4049/jimmunol.175.5.2880 16116174

[38] Kabashima K, Shiraishi N, Sugita K et al. CXCL12-CXCR4 engagement is required for migration of cutaneous dendritic cells. Am J Clin Pathol 2007; 171: 1249–57. 10.2353/ajpath.2007.070225 PMC1988874 17823289

[39] Rawat K, Tewari A, Li X et al. CCL5-producing migratory dendritic cells guide CCR5+ monocytes into the draining lymph nodes. J Exp Med 2023; 220: e20222129. 10.1084/jem.20222129 36946983 PMC10072223

[40] Zhang L, Wei X, Wang Z et al. NF-κb activation enhances STING signaling by altering microtubule-mediated STING trafficking. Cell Rep 2023; 42: 112185. 10.1016/j.celrep.2023.112185 36857187

[41] Iwamoto S, Iwai S-i, Tsujiyama K et al. TNF-α drives human CD14+ monocytes to differentiate into CD70+ dendritic cells evoking Th1 and Th17 responses. J Immunol 2007; 179: 1449–57. 10.4049/jimmunol.179.3.1449 17641010

[42] Chen S, Saeed AFUH, Liu Q et al. Macrophages in immunoregulation and therapeutics. Signal Transduct Target Ther 2023; 8: 207. 10.1038/s41392-023-01452-1 37211559 PMC10200802

[43] Yang H, Lee WS, Kong SJ et al. STING activation reprograms tumor vasculatures and synergizes with VEGFR2 blockade. J Clin Investig 2019; 129: 4350–64. 10.1172/JCI125413 31343989 PMC6763266

[44] Yang K, Han W, Jiang X et al. Zinc cyclic di-AMP nanoparticles target and suppress tumours via endothelial STING activation and tumour-associated macrophage reinvigoration. Nat Nanotechnol 2022; 17: 1322–31. 10.1038/s41565-022-01225-x 36302963

[45] La-Beck NM, Islam MR, Markiewski MM. Nanoparticle-induced complement activation: implications for cancer nanomedicine. Front Immunol 2021; 11: 603039. 10.3389/fimmu.2020.603039 33488603 PMC7819852

[46] Guo S, Yao Y, Tang Y et al. Radiation-induced tumor immune microenvironments and potential targets for combination therapy. Signal Transduct Target Ther 2023; 8: 205. 10.1038/s41392-023-01462-z 37208386 PMC10199044

[47] Begg AC, Stewart FA, Vens C. Strategies to improve radiotherapy with targeted drugs. Nat Rev Cancer 2011; 11: 239–53. 10.1038/nrc3007 21430696

[48] Vogelzang NJ . Continuous infusion chemotherapy: a critical review. J Clin Oncol 1984; 2: 1289–304. 10.1200/JCO.1984.2.11.1289 6387061

[49] Wang YH, Yao N, Wei KK et al. The efficacy and safety of probiotics for prevention of chemoradiotherapy-induced diarrhea in people with abdominal and pelvic cancer: a systematic review and meta-analysis. Eur J Clin Nutr 2016; 70: 1246–53. 10.1038/ejcn.2016.102 27329608

[50] Choi J, Kim G, Cho SB et al. Radiosensitizing high-Z metal nanoparticles for enhanced radiotherapy of glioblastoma multiforme. J Nanobiotechnol 2020; 18: 122. 10.1186/s12951-020-00684-5 PMC7470617 32883290

[51] Guidolin K, Zheng G. Nanomedicines lost in translation. ACS Nano 2019; 13: 13620–6. 10.1021/acsnano.9b08659 31800203

[52] Ngoune R, Peters A, von Elverfeldt D et al. Accumulating nanoparticles by EPR: a route of no return. J Control Release 2016; 238: 58–70. 10.1016/j.jconrel.2016.07.028 27448444

[53] Sindhwani S, Syed AM, Ngai J et al. The entry of nanoparticles into solid tumours. Nat Mater 2020; 19: 566–75. 10.1038/s41563-019-0566-2 31932672

[54] Ouyang B, Poon W, Zhang Y-N et al. The dose threshold for nanoparticle tumour delivery. Nat Mater 2020; 19: 1362–71. 10.1038/s41563-020-0755-z 32778816

[55] Yuan J, Khilnani A, Brody J et al. Current strategies for intratumoural immunotherapy—beyond immune checkpoint inhibition. Eur J Cancer 2021; 157: 493–510. 10.1016/j.ejca.2021.08.004 34561127

[56] Vincent MP, Bobbala S, Karabin NB et al. Surface chemistry-mediated modulation of adsorbed albumin folding state specifies nanocarrier clearance by distinct macrophage subsets. Nat Commun 2021; 12: 648. 10.1038/s41467-020-20886-7 33510170 PMC7844416

[57] Jiang X, Du B, Zheng J. Glutathione-mediated biotransformation in the liver modulates nanoparticle transport. Nat Nanotechnol 2019; 14: 874–82. 10.1038/s41565-019-0499-6 31308501 PMC7252432

[58] Du B, Yu M, Zheng J. Transport and interactions of nanoparticles in the kidneys. Nat Rev Mater 2018; 3: 358–74. 10.1038/s41578-018-0038-3

[59] Wang S, Chen Y, Wang S et al. DNA-functionalized metal–organic framework nanoparticles for intracellular delivery of proteins. J Am Chem Soc 2019; 141: 2215–19. 10.1021/jacs.8b12705 30669839 PMC8212418

[60] Wang S, McGuirk CM, d'Aquino A et al. Metal–organic framework nanoparticles. Adv Mater 2018; 30: 1800202. 10.1002/adma.201800202 29862586

[61] Sun X, Zhang Y, Li J et al. Amplifying STING activation by cyclic dinucleotide–manganese particles for local and systemic cancer metalloimmunotherapy. Nat Nanotechnol 2021; 16: 1260–70. 10.1038/s41565-021-00962-9 34594005 PMC8595610

[62] Sun X, Zhou X, Lei YL et al. Unlocking the promise of systemic STING agonist for cancer immunotherapy. J Control Release 2023; 357: 417–21. 10.1016/j.jconrel.2023.03.047 37001564 PMC10476228

[63] Ni K, Lan G, Guo N et al. Nanoscale metal-organic frameworks for x-ray activated in situ cancer vaccination. Sci Adv 2020; 6: eabb5223. 10.1126/sciadv.abb5223 33008911 PMC7852401

[64] Teplensky MH, Evangelopoulos M, Dittmar JW et al. Multi-antigen spherical nucleic acid cancer vaccines. Nat Biomed Eng 2023; 7: 911–27. 10.1038/s41551-022-01000-2 36717738 PMC10424220

