# Supplementary material for: STING agonist-conjugated metal-organic framework induces artificial leukocytoid structures and immune hotspots for systemic antitumor responses
Source: Natl Sci Rev. 2024 May 10;11(7):nwae167. doi: 10.1093/nsr/nwae167 (PMC11182667; doi:10.1093/nsr/nwae167)
Supplement: nwae167_Supplemental_File [file nwae167_supplemental_file.pdf]

Supplementary Information for

STING agonist-conjugated metal-organic framework induces artificial leukocytoid structures and immune hotspots for systemic antitumor responses

Taokun Luo[1]†, Xiaomin Jiang[1]†, Yingjie Fan[1], Eric Yuan[1], Jinhong Li[1], Langston Tillman[1], and Wenbin Lin[1, 2,*]

[1]Department of Chemistry, University of Chicago, Chicago, IL 60637, USA.

[2]Department of Radiation and Cellular Oncology and the Ludwig Center for Metastasis Research, University of Chicago, Chicago, IL 60637, USA.

*Corresponding author. Email: wenbinlin@uchicago.edu

†These authors contributed equally to this work.

Methods

Chemicals, cells, and animals

All starting chemicals for MOF synthesis were purchased from Sigma-Aldrich and used without purification. Adenosine phosphoramidite and guanosine phosphoramidite for GA synthesis were purchased from A2B Chem (USA) and Aaron Chemicals (China), respectively. GA was synthesized based on a previously reported method[63]. GA-Cy5 conjugate was purchased from AAT Bioquest. InVivoMAb anti-mouse PD-L1 (B7-H1) antibody was purchased from BioXCell. DPBS (-Mg^{2+}, Ca^{2+}) and nuclease-free water (no DEPC treated) were purchased from Thermo Fisher Scientific (USA). MC38, CT26, SCC7, and Raw264.7 cells were obtained from the American Type Culture Collection (ATCC, Rockville, MD). Human THP1-Dual™ KO-MyD88 monocytes were purchased from InvivoGen. Panc02 was provided by H. Schreiber from the Department of Pathology at University of Chicago. SCC7, Panc02, MC38 and Raw264.7 cells were cultured in DMEM medium (GE Healthcare, USA), and THP-1 and CT26 cells were cultured in RPMI-1640 medium (Corning, USA), which were all supported with 10% heat-inactivated fetal bovine serum (HI-FBS, 56 °C water bath for 30 minutes, VWR, USA), 100 U/ml penicillin G sodium and 100 μg/ml streptomycin sulphate and cultured in a humidified atmosphere containing 5% CO_2 at 37 °C. Mycoplasma was tested for all cells before use by MycoAlert detection kit (Lonza Nottingham, Ltd.). C3H, C57BL/6, and BALB/c mice (6-8 weeks) were obtained from Charles River Laboratories, Inc (USA). B6(Cg)-*Sting1*$^{tm1.2Camb}$/J mice (STING$^{-/-}$) were obtained from the Jackson Laboratory (USA). The study protocol was reviewed and approved by the Institutional Animal Care and Use Committee at the University of Chicago (ACUP-72408).

Synthesis and characterizations of MOF and GA-MOF

DBP-Hf MOF was synthesized as shown in Supplementary Fig. 1[11]. Briefly, to a 1-dram glass vial, 2 mg $HfCl_4$, 1 mg H_2DBP, 75 μL AA, and 1 mL DMF were added. The mixture was sonicated and heated in a 90 °C oven for 3 days. The resulting dark brown solid was collected by centrifugation, washed with DMF and ethanol, and stored as ethanol dispersions in the dark.

TFA-modified DBP-Hf (MOF) was then synthesized as shown in Supplementary Fig. 1[19]. DBP-Hf suspension in EtOH was washed sequentially with acetonitrile (CH_3CN) and benzene by sonication and centrifugation. Hf_{12}-DBP-Hf suspension in benzene (2 mM) and a 10-fold excess of trimethylsilyl trifluoroacetate (TFA-TMS) were stirred for 12 hours to obtain MOF. The suspension was washed with CH_3CN and EtOH sequentially and stored in EtOH for further use. GA-MOF was prepared by vortexing a mixture of MOF at a Hf concentration of 2.5 mM and 1 μg 2'3'-GA in 100 μL nuclease-free water for 15 minutes.

The concentrations of Hf were detected by an Agilent 7700x ICP-MS and analyzed using an ICP-MS Mass Hunter version 4.6 C.01.06. The crystallinity of MOF and GA-MOF was examined by PXRD on a Bruker D8 Venture diffractometer using a Cu Kα radiation source (λ = 1.54178 Å). The sizes and ζ-potentials were measured by a Malvern Nano Series ZetaSizer. The morphologies were observed by TEM on a TECNAI Spirit TEM and AFM on a Bruker V/Multimode 8 instrument.

Release profiles of GA-MOF

The concentration of GA was quantified by LC-MS on an Agilent 6540 Q-Tof MS-MS with 1290 UHPLC (5 μm Agilent C_{18} reverse phase column). GA-MOF was freshly prepared and redispersed in the same volume of 10× PBS, 1× PBS, 0.1× PBS, pH=4 buffer, and pH=7 buffer (100 μL/tube) in 1.5 mL Eppendorf tubes (3 replicates for each time point), respectively. The ep tubes were transferred onto a 37 °C heat block, and the supernatants (80 μL/tube) were collected at 0 h, 1 h, 2 h, 4 h, 8 h, 12 h, 24 h, 36 h, and 48 h by centrifugation at 14000 g. The supernatants were directly analyzed by LC-MS. The release percentages were fitted by the Hill function in Origin Lab software.

Endocytosis of GA-MOF

To study the endocytosis pathway of GA-MOF, 100 μM chlorpromazine, 270 μM nystatin, or 5 μM Rottlerin was added to Raw267.4 cells and incubated for 30 minutes to inhibit clathrin, caveolae, or macropinocytosis-mediated endocytotic pathway, respectively. Then the cells were rinsed with PBS and incubated with GA-MOF for 2 hours. The cells were then collected by trypsinization and digested with 1 mL of concentrated nitric acid with 1% hydrofluoric acid. The Hf content in the digest was measured by ICP-MS to calculate the cellular uptake.

X-ray irradiation

For test tube and *in vitro* experiments, an RT250 orthovoltage X-ray machine (Philips, USA) with fixed setting at 250 kVp, 15 mA, and a built-in 1 mm Cu filter was used (dose rate = 0.02564 Gy/second). For irradiating animals *in vivo*, an X-RAD 225 image-guided biological irradiator (Precision X-ray Inc., USA) was used with voltage at 225 kVp, current at 13 mA, 0.3 mm Cu filter, and 15 mm collimator (dose rate = 0.04167 Gy/second).

Growth rate inhibition assay

Cells were first seeded in 6-well plates at a density of 1×10^5 cells/well and cultured overnight. The cells were incubated with PBS or MOF (50 μM Hf) for 8 hours, and then irradiated with 0, 2, 4, or 8 Gy X-ray. The cells were washed with DPBS twice and then trypsinized to afford single-cell suspensions. The cells were counted, diluted to 1500-3000 cells/mL, and reseeded in 24-well plates. The plates were put in an IncuCyte S3 live-cell analysis system (Essen BioScience) and continuously observed by a 10× objective in phase contrast mode for up to 5 days at a 6-hour interval. The phase contrast images were collected and analyzed by IncuCyte 2021A software to

obtain time-dependent confluence in each well (25 tiles per well, 6 replicates for each treatment group). The first derivative was calculated based on a time-dependent growth curve to give a growth rate (k_t). The time point where the k of the control group (PBS, 0 Gy) reached the maximum (t_{max}) was selected as the time for the definition of GRI [64]. The GRI in radiosensitization experiment with fixed concentration of MOF is defined as the equation below:

$$GRI(D, t_{max}) = 2^{k(D,t_{\max})/k(0)} - 1$$

Where D $k(0)$ is the growth rate of the control group at t_{max}. GRI for X-ray-treated cells always falls in the range of 0 to 1, which means partial proliferation inhibition. $GRI = 0$ means complete cytostasis, and $GRI = 1$ means no cell growth inhibition. The GRI data were fitted with a linear model[26]:

$$GRI(D) = \alpha \cdot D$$

Where α is the slope. Then the growth inhibition factor at $GRI = x\%$ ($GIF_{x\%}$) was defined based on the fitting curve of $GRI(D)$ as the equation shown below:

$$GIF_{x\%} = \frac{D_{PBS}}{D_{MOL}}$$

Where D_{PBS} and D_{MOL} are the X-ray doses required to produce the same effect of $x\%$ GRI. $GRI =$ 10% was used for $GIF_{10\%}$ to quantify the radiosensitizing effect of MOF.

Immunofluorescence microscopy

All antibodies used in immunofluorescence experiments were purchased from Cell Signaling Technology except anti-mouse CD11c-PE/Cy5.5 (Invitrogen). Acti-stain™ 488 was purchased from Cytoskeleton, Inc. Hoechst 33342, and ProLong™ glass antifade mountant was purchased from ThermoFisher Scientific. The cells were seeded in 6-well plates or 12-well plates with a coverslip at the bottom of each well. The cells were treated with different conditions and fixed with 4% paraformaldehyde (pH = 7.2) at room temperature for 20 minutes. For intracellular staining, the cells were washed with DPBS, blocked, and permeabilized by 5% FBS + 0.3% Triton-X in DPBS at room temperature for 1 hour. After blocking the cells were incubated with primary antibodies in 1% BSA + 0.3% Triton-X in DPBS at 4°C overnight (phospho-STING (Ser365) (D1C4T) rabbit mAb #62912, 1:200). The cells were then washed by DPBS and incubated with secondary antibodies in 1% BSA + 0.3% Triton-X in DPBS at room temperature for 1 hour (anti-rabbit IgG (H+L), F(ab')2 fragment (Alexa Fluor® 647 conjugate) #4414, 1:1000). For staining of surface markers, the cells were blocked by DPBS with 5% FBS and stained with dye-conjugate antibody 1:100 in DPBS with 1% BSA. Both cells were washed by DPBS and further incubated with 1:500 Acti-stain™ 488 and 1:3000 Hoechst 33342 in DPBS for 30 min. After washing with DPBS, the coverslips were mounted on glass slides with ProLong™ glass antifade mountant, cured at 4°C overnight, sealed by nail polish, and observed on a Leica Stellaris 8 confocal microscope. The data were analyzed with Fiji ImageJ (NIH).

Isothermal titration calorimetry

The interaction between GA and MOF was analyzed on a MicroCal iTC$_{200}$ system (Malvern Instruments) equipped with reference and sample cells (V = 400 μL). All titrations were carried out using a 40 μL syringe at 298.15 K with a stirring rate of 250 rpm. The aqueous dispersion of MOF (900 μM Hf) was titrated with 300 μM GA aqueous solution. A first injection of 0.4 μL was followed by 20 injections of 2 μL at intervals of 150 s. Data analysis was performed using the

MicroCal iTC_{200} software, and all data were fitted to an independent single-site model.

Bone marrow-derived dendritic cells and macrophages

6 to 8-week-old female C57BL/c mice were euthanized, and bone marrow cells were flushed out from the femur and tibia using insulin syringes with RPMI-1640. Red blood cells were lysed by sterile ACK buffer (Corning), and the rest of the cells were cultured in RPMI-1640 full medium + 20 ng/mL recombinant mouse granulocyte-macrophage colony-stimulating factor (GM-CSF, R&D Systems) + 10 ng/mL recombinant murine interleukin-4 (IL-4, PeproTech). On day 2, half of the medium was discarded, and fresh prewarmed medium with 40 ng/mL GM-CSF and 20 ng/mL IL-4 was added. On day 4, the entire medium was discarded and replaced by fresh and warm medium with 20 ng/mL GM-CSF and 10ng/mL IL-4. On day 8, the semi-suspended and loosely attached cells were collected by gently pipetting, and the medium suspension was collected as BMDCs. The adherent cells were gently scraped off by cell scrapers as BMDMs. The purity of the cells was confirmed by flow cytometry with CD11c-PE/Cy5.5 (N418) and F4/80-PerCP/Cy5.5 (BM8) antibodies, respectively. The phosphorylated STING and IRF-3 were also stained and visualized for BMDCs and Raw264.7, respectively.

STING activation *in vitro*

THP1-Dual™ KO-MyD88 reporter cells were used to quantify STING activation by GA and GA-MOF *in vitro*. The cells were seeded in 96-well plates at a density of 2×10^5 cells/mL (N=3), and up to 139.2 μM GA or GA-MOF was added and incubated for 24 hours. The stimulation of the IRF pathway was quantified by QUANTI-Luc (InvivoGen) assay on a Synergy HTX plate reader. The cytokines secreted in the medium were quantified by LumiKine™ Xpress mIFN-β 2.0 (InvivoGen), TNFα mouse ELISA kit, and IL-6 mouse ELISA kit (Invitrogen).

Phagocytosis

In 6-well plates with collagen-coated coverslips, BMDCs or BMDMs (1 mL RPMI-1640 full medium, 500,000 cells/well) were seeded and incubated at 37 °C. Eight hours later, CFSE-labeled MC38 cells (1 mL RPMI-1640 full medium, 250,000 cells/well) were added together with GA (0.1 μg/mL), MOF (25 μM Hf), or GA-MOF (0.1 μg/mL GA and 25 μM Hf). Four hours later, the plates were irradiated with 2 Gy X-ray and incubated for another 24 hours. The cells were then fixed by 4% PFA and stained with CD11c-PE/Cy5.5 (N418) or F4/80-PerCP/Cy5.5 (BM8) to visualize BMDCs or BMDMs with a Leica Stellaris 8 confocal microscope, respectively.

Cell viability assays

The cytotoxicity of MOF and GA-MOF to immune cells was evaluated in splenocytes, BMDMs, and BMDCs by CellTiter 96 AQ_{ueous} One Solution Cell Proliferation Assay (MTS assay, Promega, USA). The cells were seeded in 96-well plates at a density of 10,000 cells/well and cultured overnight. MOF or GA-MOF was added at a Hf concentration of 0, 0.2, 0.4, 0.8, 1.6, 3.1, 6.3, 12.5, 25, 50, and 100 μM and incubated for 8 hours. The cells were then irradiated with 0-8 Gy X-ray and allowed to incubate for another 3 days (N=3). The cell viability was determined by MTS assay.

Pharmacokinetics of GA-MOF in rats

SD/CD female rats (6 weeks, 160–200 g) were anesthetized and subcutaneously injected with GA-MOF. The rats were anesthetized at 5 min, 30 min, 1 h, 2 h, 4 h, and 24 h, and the blood was drawn from the retro-orbital plexus. The whole blood was immediately centrifuged at 2,000 g at 4 °C for 15 minutes. The clear top layer was immediately transferred, aliquoted into 1.5 mL ep tubes, and

tested by GA ELISA Kit (Cayman Chemical) for GA concentration.

GA retention in tumors

6~8-week-old CT26-bearing BALB/c mice were anesthetized and *i.t.* injected with GA-MOF in 20 μL saline. The mice were anesthetized at 5 min, 30 min, 1 h, 2 h, 4 h, 24 h, 48 h, 96 h, and 168 h, and the blood was drawn by eyeball removal. The mice were then euthanized. The tumors were excised and kept on ice. Each tumor was then immersed in an individual 15 mL ep tube filled with 2 mL 10x PBS (on ice). The tumor tissues were then homogenized with a probe sonicator (500 W, 20 kHz) with 30% power for 1 minute on ice. The mixture was centrifuged at 4 °C at 14,000 g for 15 minutes. The supernatants were collected, aliquoted into 1.5 mL ep tubes, and tested by GA ELISA Kit (Cayman Chemical) for GA concentration.

***In vivo* antitumor efficacy**

The antitumor efficacy of GA-MOF was evaluated on subcutaneous CT26 tumor-bearing BALB/c, MC38 tumor- and Panc02 tumor-bearing C57BL/6, and SCC7 tumor-bearing C3H mouse models. For the single tumor model, 2×10^6 CT26 cells, SCC7 cells, MC38 cells, and Panc02 cells were subcutaneously injected into the right flanks. When the tumors reached 75-100 mm^3, MOF (0.5 μmol Hf), GA-MOF (0.5 μmol Hf, 2 μg GA), GA (2 μg GA), or PBS was *i.t.* injected into the mice. Eight hours later, the mice were anesthetized with 2.5 % (v/v) isoflurane/oxygen, and the tumors were irradiated with X-ray at the following doses: CT26, 2 Gy × 3 (N=8); MC38, 4 Gy × 3 (N=7); Panc02, 3 Gy × 3 (N=5); SCC7, 3 Gy × 3 (N=6). For the immune cell depletion experiment, 250 μg neutralizing antibody was given intraperitoneally (*i.p.*) to MC38 tumor-bearing C57BL/6 mice four days after tumor inoculation, followed by *i.p.* injection of neutralizing antibodies for 3 consecutive days, then continued with *i.p* injection once per week until the study endpoint. The tumor volumes (length×width^2/2), body weights, and health conditions of the mice were monitored, and the mice were euthanized according to the protocol limit. The tumor growth inhibition index (TGI) was calculated as the equation below:

$$TGI = 1 - \frac{\frac{T_e}{T_s} \Big/ \frac{C_e}{C_s}}{1 - \frac{C_s}{C_e}} \times 100\%$$

Where T_e, T_s, C_e, and C_s represent average tumor volumes of treated mice at the endpoint, treated mice at the starting point, control mice at the endpoint, and control mice at the starting point, respectively.

Intratumoral cytokine levels

6~8-week-old MC38 tumor-bearing C57BL/6 mice were treated in the same way as the efficacy study. Three days after the last RT dose, the mice were euthanized, and the tumors were excised and kept on ice. Each tumor was then immersed in an individual 15 mL ep tube filled with 2 mL RIPA buffer (on ice). The tumor tissues were homogenized with a probe sonicator (500 W, 20 kHz) with 30% power for 30 seconds on ice. The mixture was centrifuged at 4 °C at 14,000 g for 15 minutes. The supernatants were collected, aliquoted into 1.5 mL ep tubes, and tested by respective ELISA kits (Invitrogen) for cytokine concentrations.

Abscopal effect

The abscopal effect of GA-MOF plus αPD-L1 was evaluated in two bilateral subcutaneous mouse

models. For the bilateral CT26 or MC38 tumor model, 2 × 10^6 CT26 or MC38 cells were subcutaneously injected into the right flanks, and 8 × 10^5 CT26 or MC38 cells were injected into the left flanks of BALB/c or C57BL/6 mice, respectively (*N*=6). When the primary tumors (right) reached 100-125 mm^3, the primary tumors were injected with GA-MOF, GA, or PBS. The mice received X-ray treatment on the primary tumors at 2 Gy × 3 for CT26 and 4 Gy × 3 for MC38. The CT26 model in αPD-L1, GA + αPD-L1, or GA-MOF + αPD-L1 group was *i.p.* injected with 75 μg/mouse αPD-L1 on day 3 and 6 after the first X-ray treatment. The MC38 model in αPD-L1, free GA + αPD-L1, or GA-MOF + αPD-L1 group was *i.p.* injected with 100 μg/mouse αPD-L1 on day 2, 4, and 6 after the first X-ray treatment. The tumor volumes, body weights, and health conditions of the mice were monitored, and the mice were euthanized according to the protocol limit.

Immune cell profiling

MC38 tumor-bearing C57BL/6 mice (*N*=6) received *i.t.* injections and X-ray doses, and the tumors and tumor-draining lymph nodes (TDLNs) were harvested on day 13 or day 25 for immune cell profiling by flow cytometry. The tumors and TDLNs were digested by RPMI-1640 + 10% FBS + 0.5 mg/mL collagenase I (Gibco) + 200 μg/mL collagenase IV (Gibco)+ 50 μg/mL Dnase I (Sigma-Aldrich) cocktail at 37 °C for 45 minutes. The digests were gently ground and filtered through sterile cell strainers (40 μm, Corning) to obtain single-cell suspensions. The cells were washed by ice-cold FACS buffer and stained first with eFluor™ 506-Fixable Viability Dye (ThermoFisher Scientific, 1:1000). The cells were then washed with FACS buffer, blocked by anti-CD16/32 antibody (clone 93, 1:100) at 4 °C for 15 minutes, and stained with the following fluorochrome-conjugated rat anti-mouse antibodies 1:200 (1:500 for CD45-BV421) at 4 °C for 45 minutes: CD45-BV421 (30-F11), CD45-AlexFluor488 (30-F11), CD45-PacificBlue (30-F11), CD11b-SB600 (M1/70), CD11b-FITC (M1/70), NK1.1-PE/Dazzole594 (PK130), F4/80-PerCP/Cy5.5 (BM8), Gr-1-PE (RB6-8C5), Gr-1-APC/eFluor780 (RB6-8C5), CD86-PE (GL1), CD206-APC (C068C2), CD11c-PE/Cy5.5 (N418), CD11c-BV421 (N418), MHCII-PE (M5/114.15.2), MHCII-PE/Cy7 (M5/114.15.2), CD3-PE/eFluor610 (145-2C11), CD3-PE/Cy7 (145-2C11), CD3-SB600 (145-2C11), CD4-APC/H7 (GK1.5), CD4-AlexaFluor488 (GK1.5), CD8-PerCP/eFluor710 (53-6.7), CD8-APC/eFluor780 (53-6.7), B220-APC (RA3-6B2), B220-PE/Cy5 (RA3-6B2), CD44-PE (IM7), CD62L-FITC (MEL-14), CD62L-APC (MEL-14), Tbet-PE/Cy5 (4B10), PD1-PE/Cy7 (J43), FoxP3-APC (FJK-16s), CD25-PE (12-0251-82). CD45-BV421 was from BD Bioscience. CD206-PE/Cy7, NK1.1-PE/Dazzole594 and CD3-PE/Cy7 were from BioLegend. Others were from eBioscience. The cells were finally washed and resuspended in FACS buffer and analyzed on an LSR Fortessa 4-15 flow cytometer.

NanoString analysis

SCC7 tumors were digested in the same way as immune cell profiling to afford single-cell suspensions of tumor cells. The RNA was extracted and purified with the Rneasy Mini Kit (QIAGEN). The RNA concentration was normalized to 20 ng/μL in nuclease-free water with a NanoDrop Eight spectrophotometer (Thermo Fisher Scientific). The RNA was then hybridized with the Gene Expression CodeSet of the nCounter PanCancer Immune Profiling Panel (NanoString) in a thermal cycler (Bio-Rad), and processed and imaged with the nCounter MAX/FLEX system (NanoString). The data was analyzed with ROSALIND.

Immunohistochemistry analysis

For the formalin-fixed-paraffin-embedded (FFPE) samples, the tumors and major organs were

harvested from the treated mice, washed with DPBS, fixed with 10% neutral buffer (4% PFA) for 24 hours and 70% ethanol for 24 hours. The tissues were processed, embedded in paraffin, sectioned, and stained (H&E, IBA-1, CD3e) by the Human Tissue Resource Center at the University of Chicago. Briefly, the slides were deparaffinized and rehydrated through xylenes and serial dilutions of ethanol to distilled water. Then the slides were treated with antigen retrieval buffer (S1699, DAKO for CD3e; S2367, DAKO for IBA-1) and heated in a steamer over 97°C for 20 minutes). After washing, the slides were incubated with primary antibodies (anti-CD3, Abcam (ab5690), 1:100; anti-IBA-1, Cell Signaling (E4O4W), 1:600) at room temperature for 1 hour in a wet chamber. The slides were washed with TBS, then CD3 and IBA-1 slides were incubated with biotinylated anti-rabbit IgG (1:200, BA-1000, Vector Laboratories) and anti-rabbit-polymer (Bond Polymer refine Detection, Leica Biosystems, DS9800) at room temperature for 30 minutes, respectively. The antigen-antibody binding was detected by Elite kit (PK-6100, Vector Laboratories) and DAB (DAKO, K3468) system. Tissue sections were then immersed in hematoxylin for counterstaining and e covered with cover glasses. The slides were scanned on a Cri Pannoramic SCAN 40x whole slide scanner by Integrated Light Microscopy Core at the University of Chicago. The images were analyzed by QuPath-0.4.2 software[65].

IFN-γ ELISPOT assay

A Multiscreen HTS-IP plate (Millipore Sigma) was activated by 70% ethanol, washed with DPBS, coated with anti-mouse IFN-γ capture antibody (BD Biosciences) at 37 °C for 8 hours, and blocked with sterile 1% BSA in DPBS at room temperature for 2 hours. The spleens were harvested from the treated bilateral CT26 tumor-bearing BALB/c and MC38 tumor-bearing C57BL/6 mice, and then gently grinded and filtered through sterile cell strainers to afford single cell suspensions. Red blood cells were then lysed by sterile ACK buffer (Corning), and splenocytes were counted and seeded in the plate at a density of 2×10^5 cells/well in RPMI-1640 full medium (6 mice each treatment group and each mouse with 3 replicates). CT26 tumor-associated antigen SPSYVYHQF (AH1) or MC38 tumor-associated antigen KSPWFTTL (KSP) was added to each well at a concentration of 10 μg/mL except for negative control wells. The splenocytes in positive control wells were directly stimulated with anti-mouse CD3Ɛ (145-2C11) and anti-mouse CD28 (37.51) antibodies (eBioscience, 1:1000). The splenocytes were incubated at 37 °C for 48 hours and culture media were discarded. The plates were then washed and incubated with biotinylated anti-IFN-γ detection antibody, streptavidin-HRP conjugate, and AEC substrate following the manufacturer's specification (BD Biosciences). The plate was air-dried and analyzed by a CTL ImmunoSpot® S6 Analyzer.

Biodistribution

To evaluate biodistribution of GA-MOF, a subcutaneous MC38 model was established in C57BL/6 mice by inoculating 2×10^6 MC38 cells/mouse subcutaneously. At day 7, GA-MOF was injected intratumorally with an equivalent Hf dose of 0.5 μmol. At day 14 and day 21, the mice were anesthetized ($N = 3$), and the major organs were excised and digested by concentrated HNO_3. The Hf content was then quantified by ICP-MS. Only two mice at each time point were used in biodistribution analysis due to the statistical outlying nature of the third datapoint.

Statistical analysis

The statistical analysis was performed on Origin Lab software by paired-sample *t*-test or One-way Repeated Measures ANOVA methods with Tukey's honest significance test.

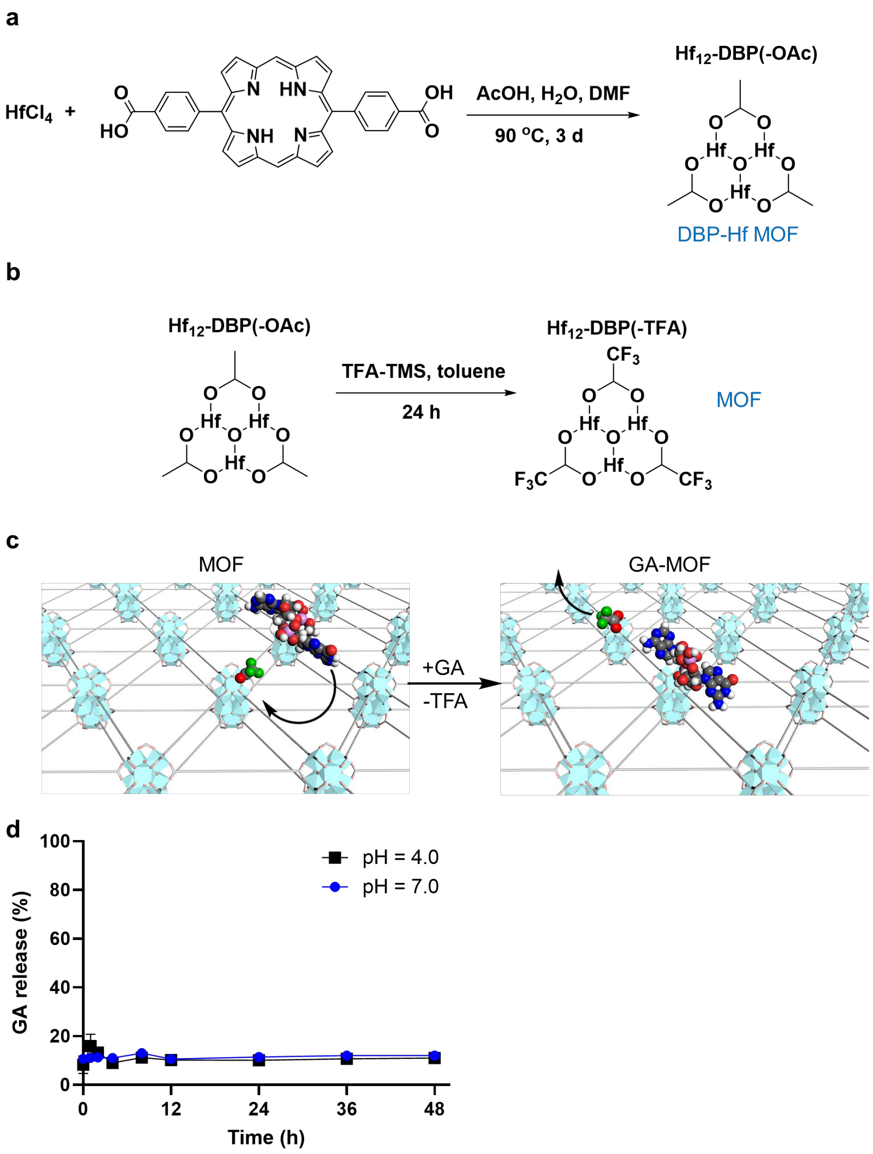

Supplementary Fig 1. Synthesis of DBP-Hf, TFA-modified Hf-DBP, and GA-MOF

(a) Solvothermal synthesis of Hf-DBP nanoscale MOF. AcOH, acetic acid. (b) TFA replacement of acetate groups (OAc) on Hf_{12} SBUs by TFA-TMS in anhydrous toluene to afford TFA-modified MOF. (c) Substitution of TFA by phosphate groups of GA to afford GA-MOF. Cyan polyhedron, Hf; Grey stick, DBP ligand; Grey sphere, C. Green sphere, F; Blue sphere, N; Red sphere, O; Pink sphere; P. White sphere, H. (d) GA release from GA-MOF at pH=4 or pH=7 measured by LC-MS (*N*=3).

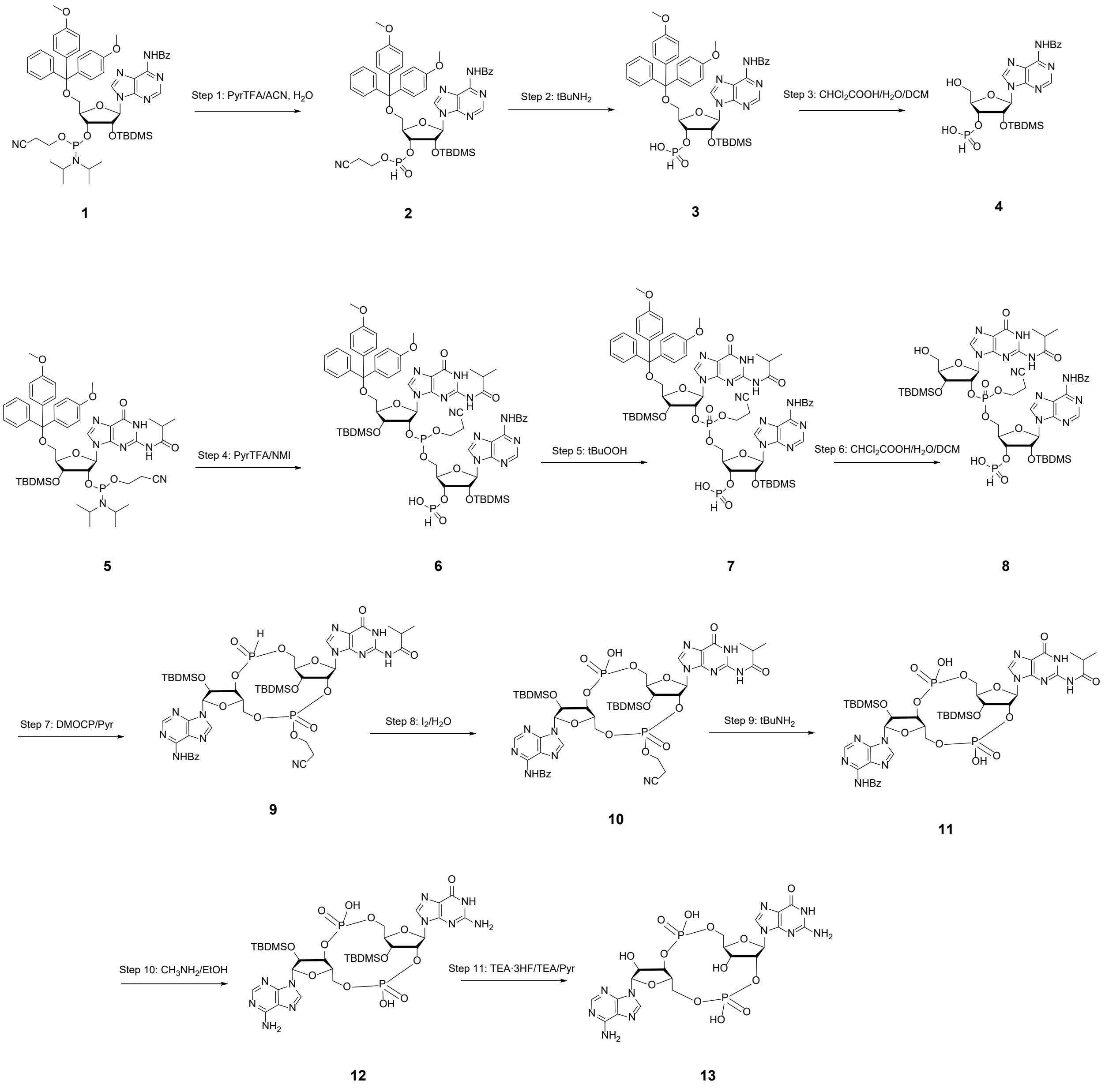

Supplementary Fig 2. Synthesis of GA

The synthetic route of GA.

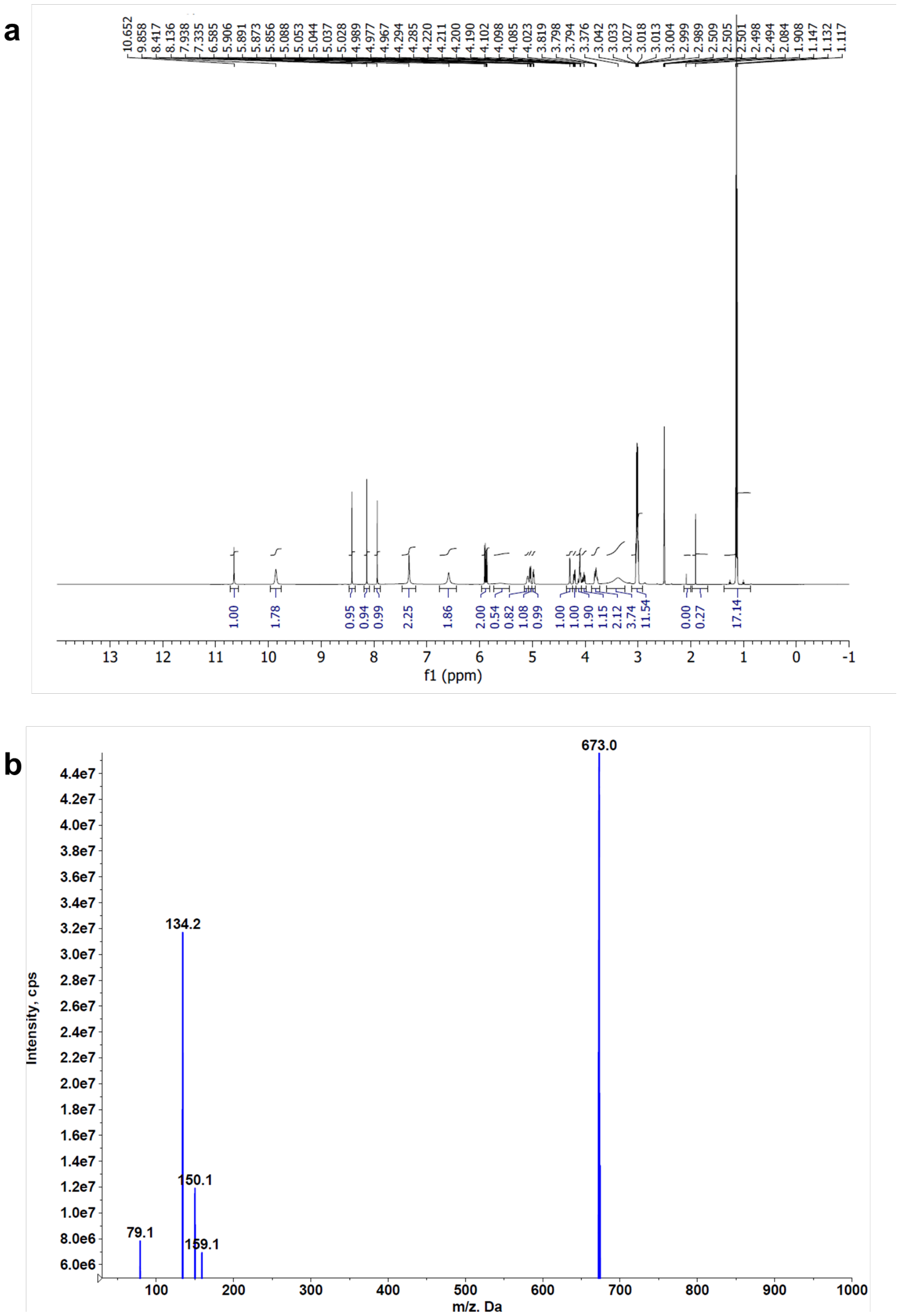

Supplementary Fig 3. Characterization of GA

(a) ^{1}H NMR of GA (500 MHz, DMSO-d$_6$): δ 10.65 (s, 1H), 9.86 (bs, 2H), 8.42 (s, 1H), 8.14 (s, 1H), 7.94 (s, 1H), 7.34 (bs, 2H), 6.59 (bs, 2H), 5.90 (d, *J* = 7.5 *Hz*, 1H), 5.86 (d, *J* = 7.5 *Hz*,, 1H), 5.09 (bs, 1H), 5.05–5.03 (m, 1H), 4.99–4.97 (m, 1H), 4.29 (d, *J* = 4.5 *Hz*, 1H), 4.22–4.19 (m, 1H), 4.10–4.02 (m, 3H), 3.82–3.79 (m, 2H), 3.74 (bs, 2H), 3.04–2.99 (m, 12H), 1.13 (t, *J* = 7.5 *Hz*, 18H). (b) ESI$^-$ Mass spectroscopy of GA. MS (ESI$^-$) m/z = 673.1 [M-H]$^-$, found 673.0 m/z.

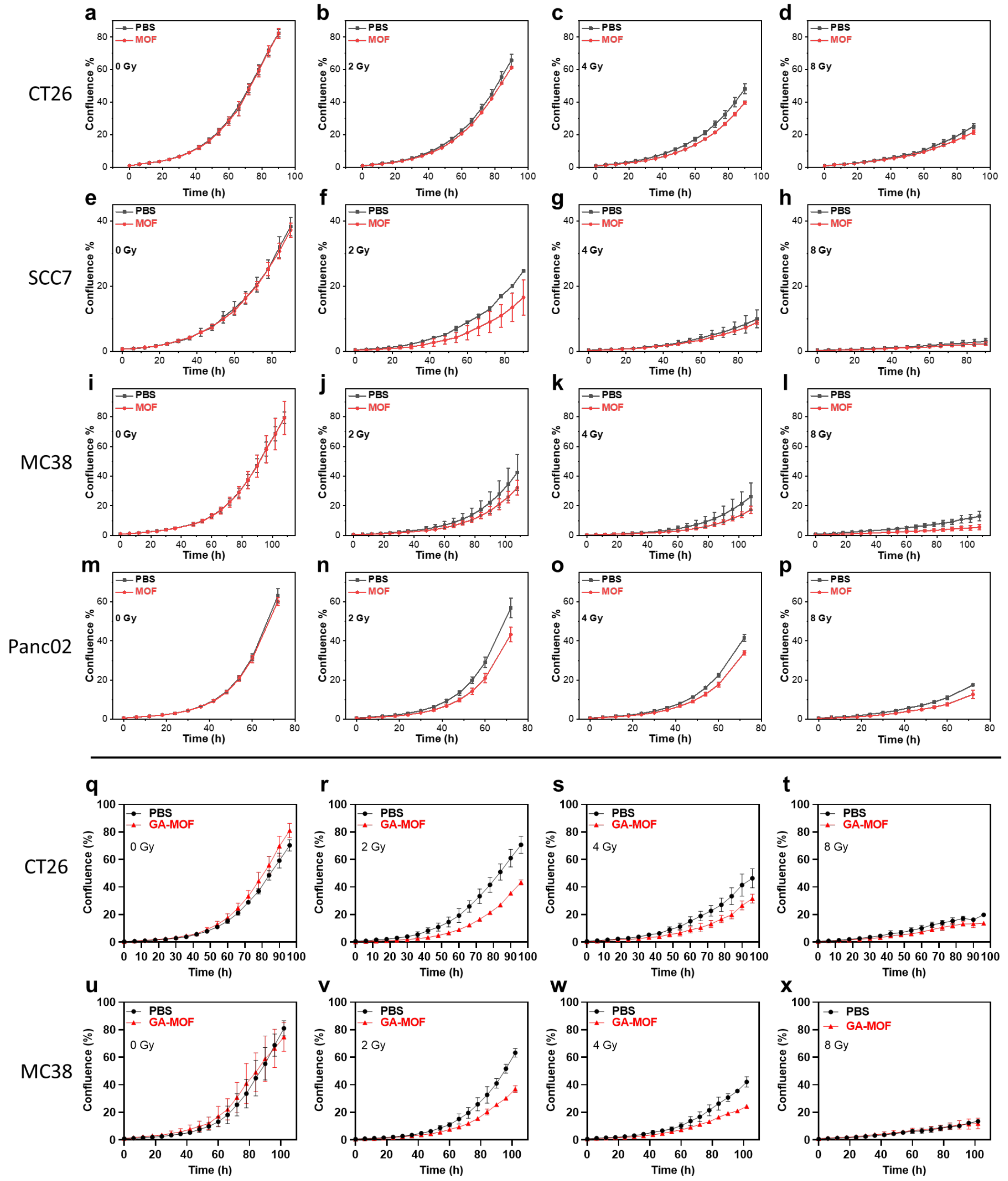

Supplementary Fig 4. Time-dependent confluence curves in GR assays

(a-d) Real-time confluence of CT26 cells treated by MOF and X-ray irradiation from 0 (a), 2 Gy (b), 4 Gy (c) to 8 Gy (d). (e-h) Real-time confluence of SCC7 cells treated by MOF and X-ray irradiation from 0 (e), 2 Gy (f), 4 Gy (g) to 8 Gy (h). (i-l) Real-time confluence of MC38 cells treated by MOF and X-ray irradiation from 0 (i), 2 Gy (j), 4 Gy (k) to 8 Gy (l). (m-p) Real-time confluence of Panc02 cells treated by MOF and X-ray irradiation from 0 (m), 2 Gy (n), 4 Gy (o) to 8 Gy (p). (q-t) Real-time confluence of CT26 cells treated by GA-MOF and X-ray irradiation from 0 (q), 2 Gy (r), 4 Gy (s) to 8 Gy (t). (u-x) Real-time confluence of MC38 cells treated by GA-MOF and X-ray irradiation from 0 (u), 2 Gy (v), 4 Gy (w) to 8 Gy (x). The data were acquired and analyzed by IncuCyte S3 live imaging system (*N*=3).

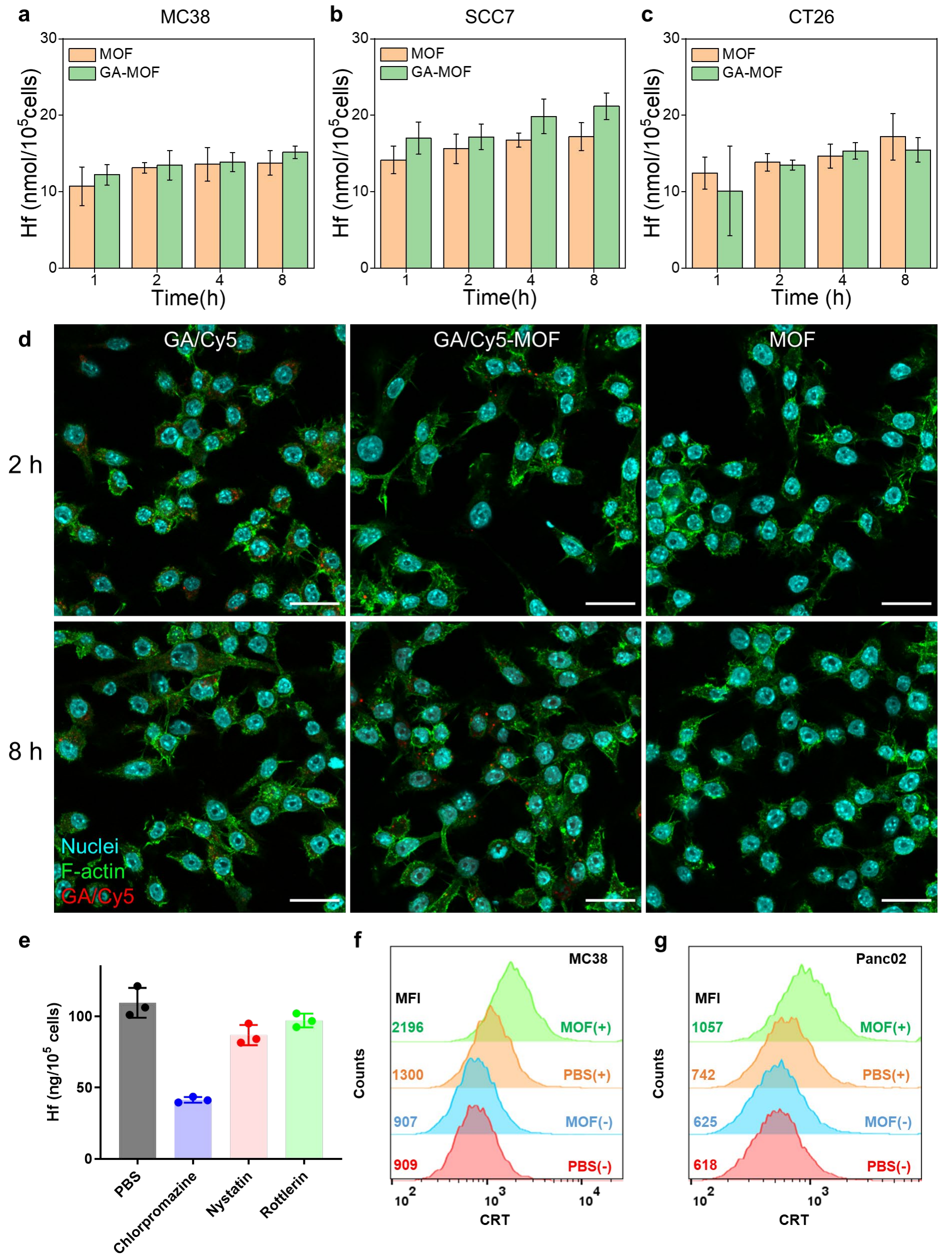

Supplementary Fig 5. Cellular uptake and i*n vitro* trafficking of GA-MOF

(a-c) Cellular uptake of MOF and GA-MOF in MC38, SCC7, and CT26 cells (N=3). (d) Representative CLSM images showing uptake of Cy5-labeled (red) GA or GA/Cy5-MOF after incubation with Raw264.7 cells for 2-8 hours. Cell nuclei are shown in cyan, and cytoskeletons are shown in green (scale bar = 10 μm). (e) Cellular uptake of GA-MOF by Raw264.7 cells after treatment with 100 μM chlorpromazine, 270 μM nystatin, or 5 μM rottlerin to inhibit clathrin, caveolae, or macropinocytosis-mediated endocytosis pathway, respectively. The experiment results indicated that Raw264.7 cells take up GA-MOF mainly through clathrin-mediated endocytosis. (f,g) Flow cytometry histograms showing CRT surface translocation of treated (f) MC38 and (g) Panc02 cells as a marker of ICD.

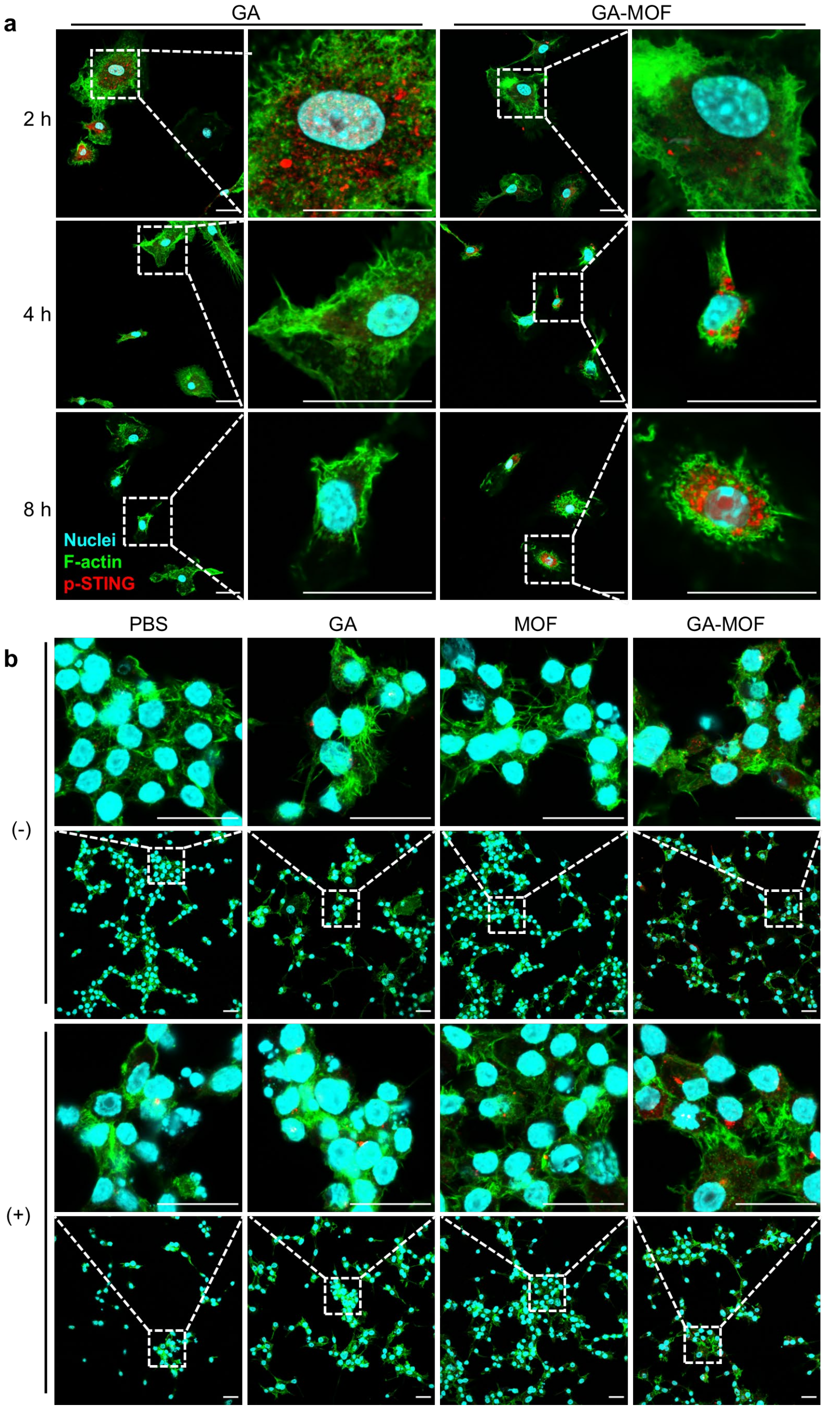

Supplementary Fig 6. ***In vitro*** **STING activation by GA-MOF**

(a) Representative CLSM images showing time-dependent expression of p-STING (red) in BMDCs treated with GA or GA-MOF. Cell nuclei are shown in cyan, and cytoskeletons are shown in green (scale bar = 20 μm). (b) Representative CLSM images showing p-IRF-3 (red) upregulation in Raw264.7 cells treated with PBS, GA, MOF, or GA-MOF with (+) or without (-) 2 Gy X-ray irradiation. Cell nuclei are shown in cyan, and cytoskeletons are shown in green (scale bar = 10 μm).

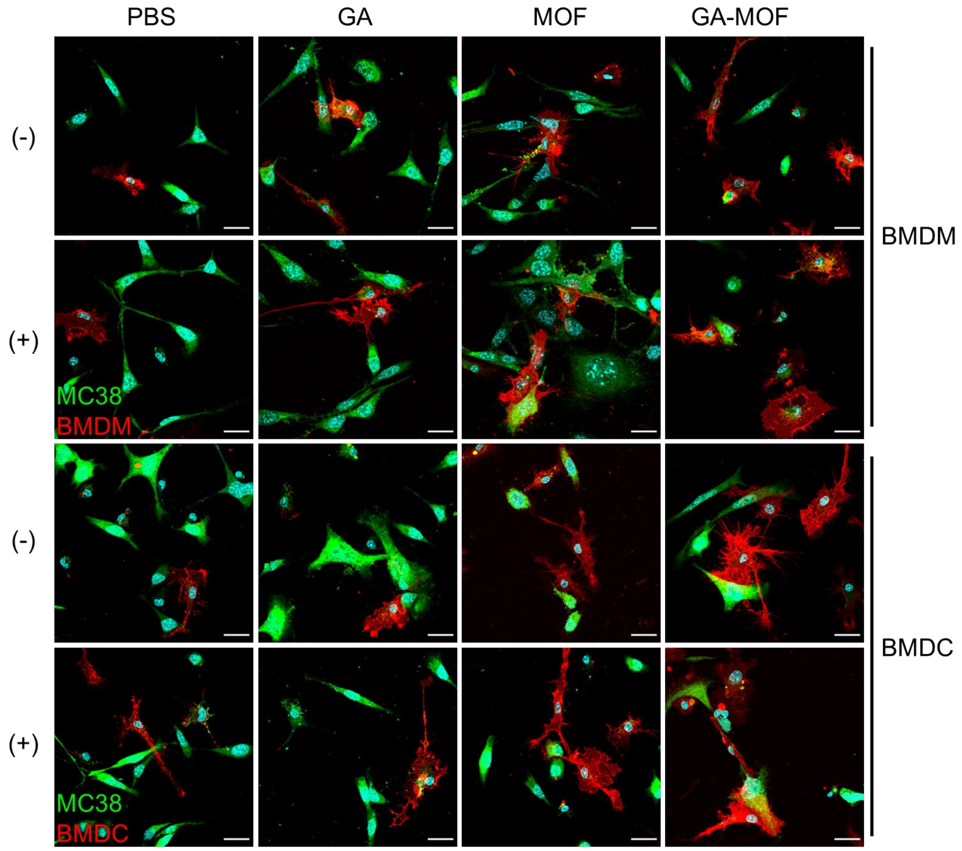

Supplementary Fig 7. Phagocytosis stimulated by GA-MOF

Representative CLSM images showing CFSE-labeled MC38 cells (green) phagocytized by F4/80-labeled BMDMs (red) or CD11c-labeled BMDCs (red) after different treatments (scale bar = 20 μm). (+) denotes 2 Gy X-ray irradiation 4 hours post particle incubation. (-) denoted no irradiation treatment.

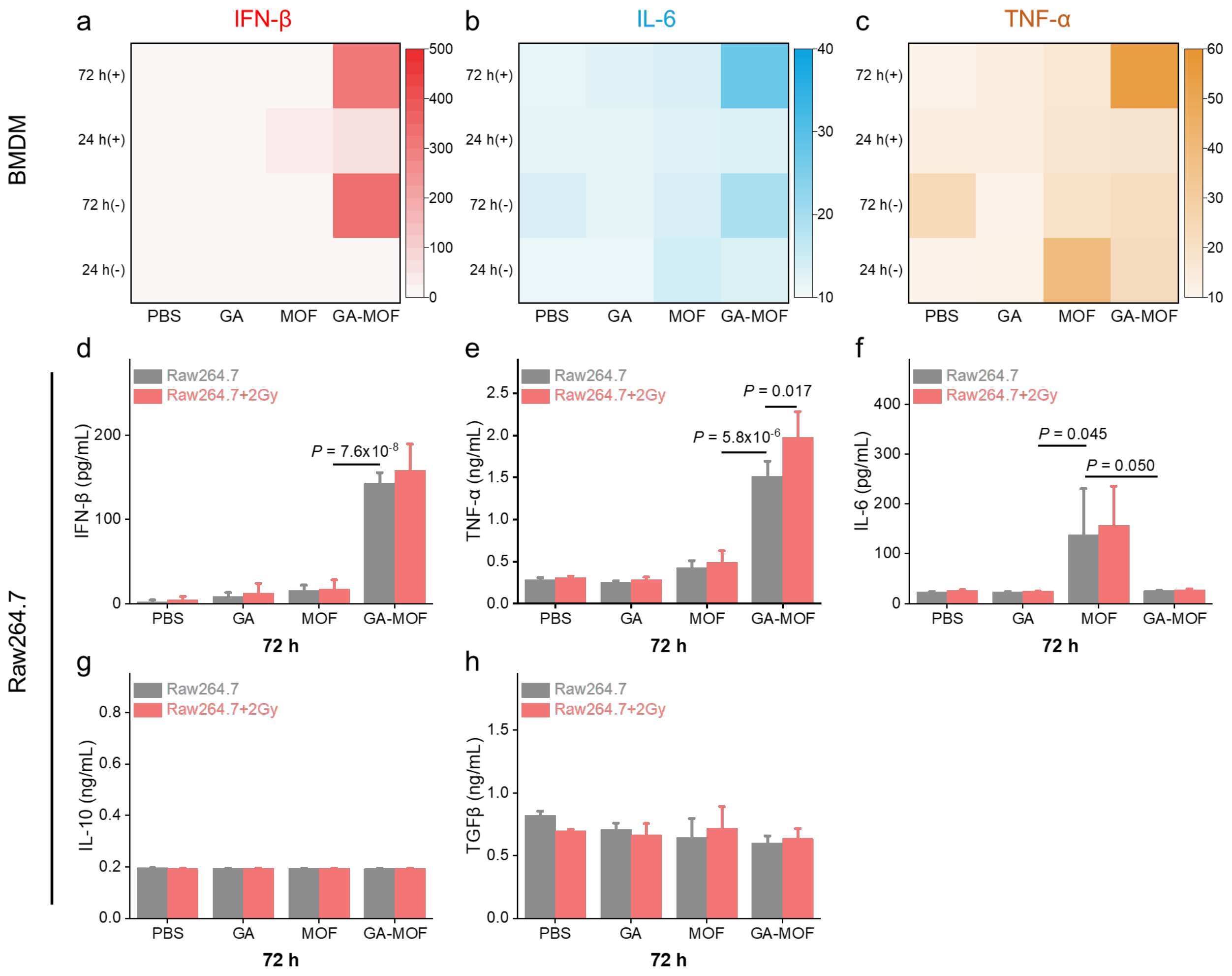

Supplementary Fig 8. Cytokine secretion stimulated by GA-MOF *in vitro*

(a-c) Heat maps showing secretion levels of IFN-β (a), IL-6 (b), and TNF-α (c) by BMDMs (N=3). In each figure, the concentration of the cytokine was given as pg/mL. (+) denotes 2 Gy X-ray irradiation 4 hours post particle incubation. (-) denoted no irradiation treatment. The x-axis showed treatment groups, and the y-axis showed incubation time and whether 2 Gy X-ray was given (+) or not (-). (d-h) Bar graphs showing secretion levels of IFN-β (d), IL-6 (e), TNF-α (f), IL-10 (g), and TGFβ (h) by Raw264.7 cells after different treatments with or without 2 Gy X-ray irradiation (N=3). The incubation time is listed under the y axis.

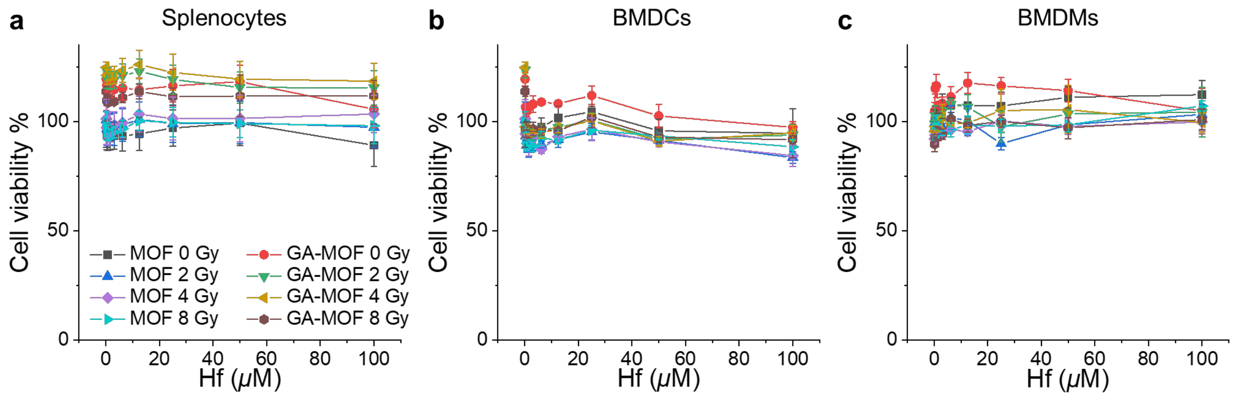

Supplementary Fig 9. Cytotoxicity of MOF and GA-MOF on immune cells

(a-c) Cell viabilities of splenocytes (a), BMDCs (b), and BMDMs (c) after MOF or GA-MOF incubation (up to an equivalent concentration of 100 μM Hf) for 3 days with 0, 2, 4, or 8 Gy X-ray irradiation by MTS assay.

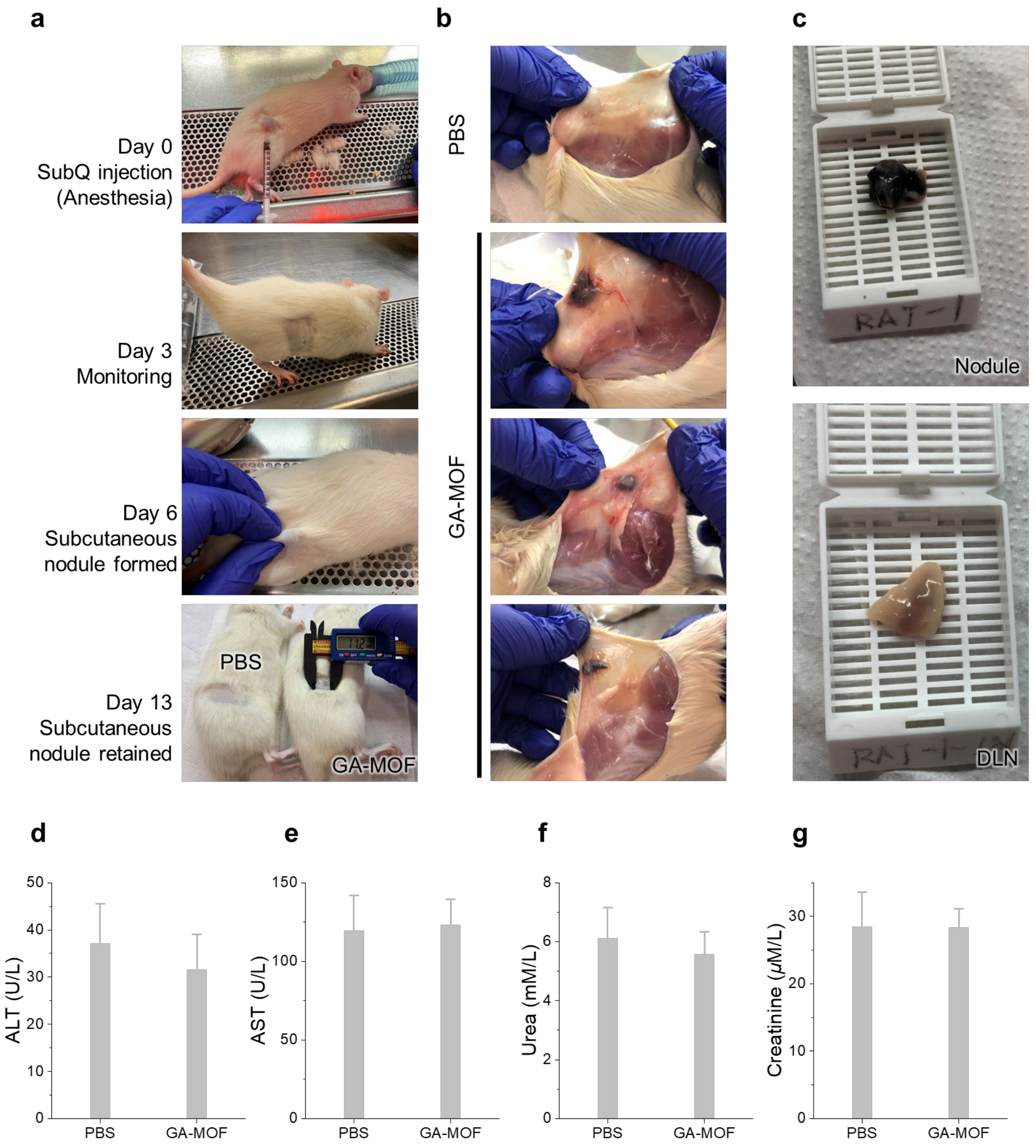

Supplementary Fig 10. Formation of subcutaneous nodules in rats treated with GA-MOF and rat liver and kidney function tests

(a) Timeline of nodule formation after subcutaneous injection of GA-MOF into SD/CD rats. (b) Anatomical observation of the subcutaneous space after injection of PBS or GA-MOF (*N*=3 for the GA-MOF group). (c) Photos of dissected subcutaneous nodules (top) and the draining LNs (bottom). (d-g) Alanine transaminase (ALT, d), aspartate aminotransferase (AST, e), urea (f), and creatinine (g) levels in rat plasmas after two subcutaneous injections of GA-MOF on day 1 and day 8 (*N*=10). The plasma samples were collected for liver and kidney function tests on day 15.

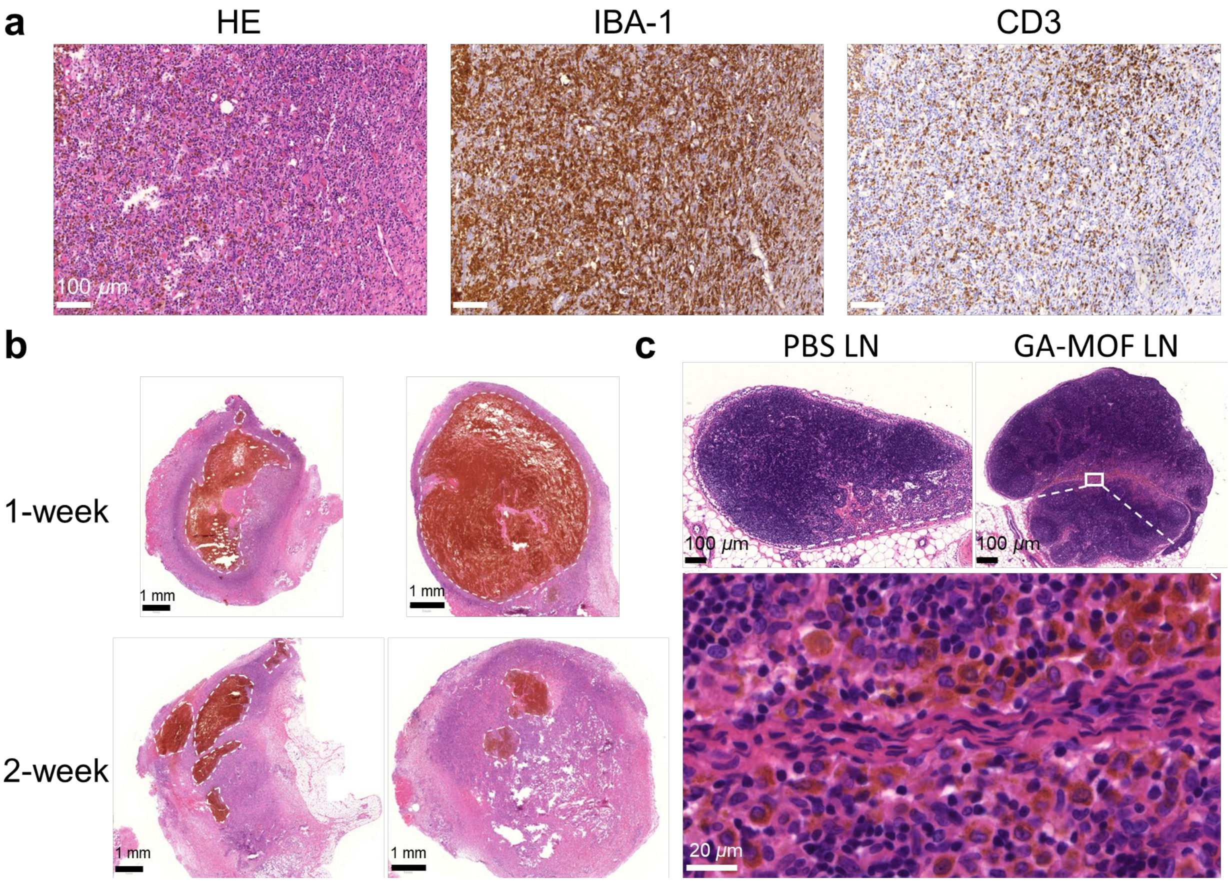

Supplementary Fig 11. Histological staining of subcutaneous nodules

(a) HE (left), IBA-1 (middle), and CD3 (right) staining showing the nodule morphology, infiltration of innate immune cells, and adaptive immune cells, respectively (scale bar = 100 μm). (b) HE staining of the nodules one week (top) and two weeks (bottom) post injection (scale bar = 1 mm, MOF contents are circled with white dashed lines). (c) Draining LNs of PBS- and GA-MOF-treated rats (scale bar = 100 μm). The zoomed-in image for the GA-MOF group is shown at the bottom (scale bar = 20 μm).

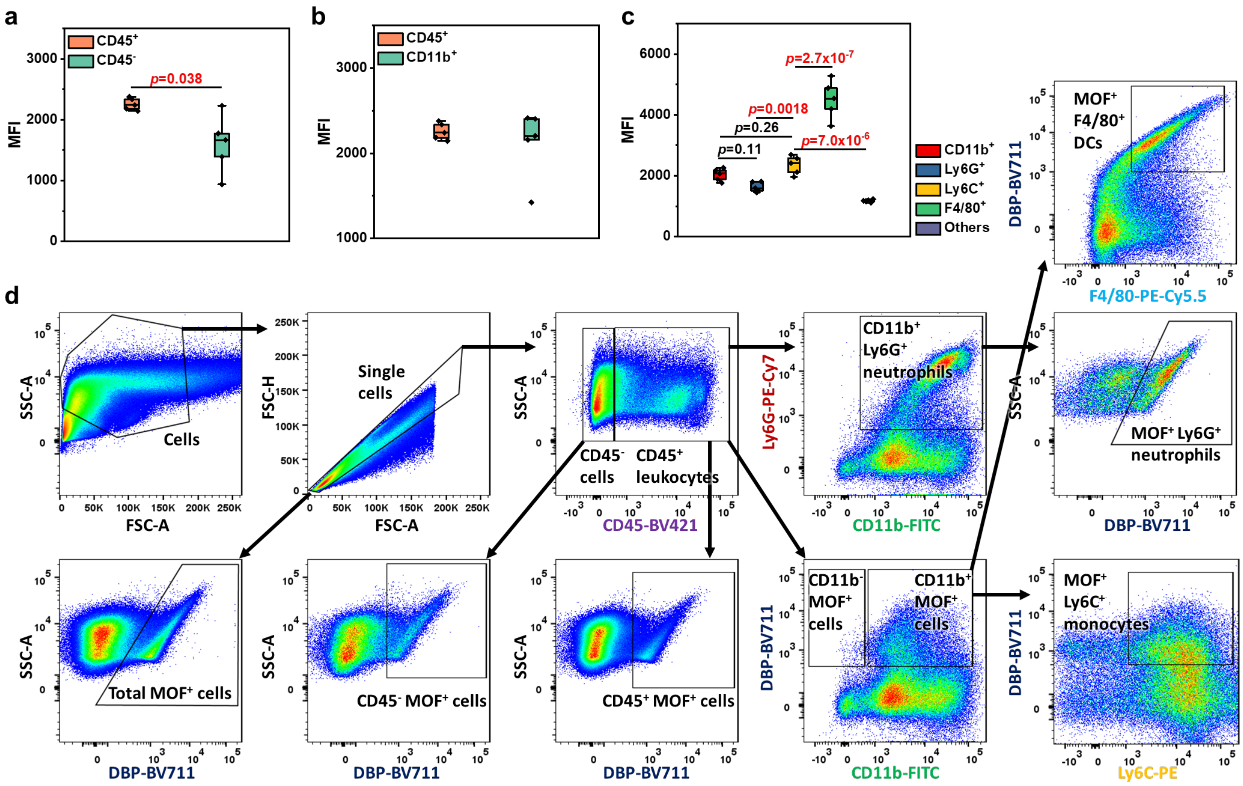

Supplementary Fig 12. Relative MOF uptake amounts and gating strategies for MOF-treated cells

(a) Mean fluorescence intensity (MFI) of MOF in CD45$^+$ leukocytes and CD45$^-$ cells (mostly cancer cells). (b) MFI of MOF in CD45$^+$ leukocytes and CD11b$^+$ myeloid cells. (c) MFI of MOF in CD11b$^+$ myeloid cells, Ly6G$^+$ cells, Ly6C$^+$ cells, F4/80$^+$ cells, and other uncategorized cells (N=5). (d) Representative gating strategies for MOF-uptake cells among different immune populations.

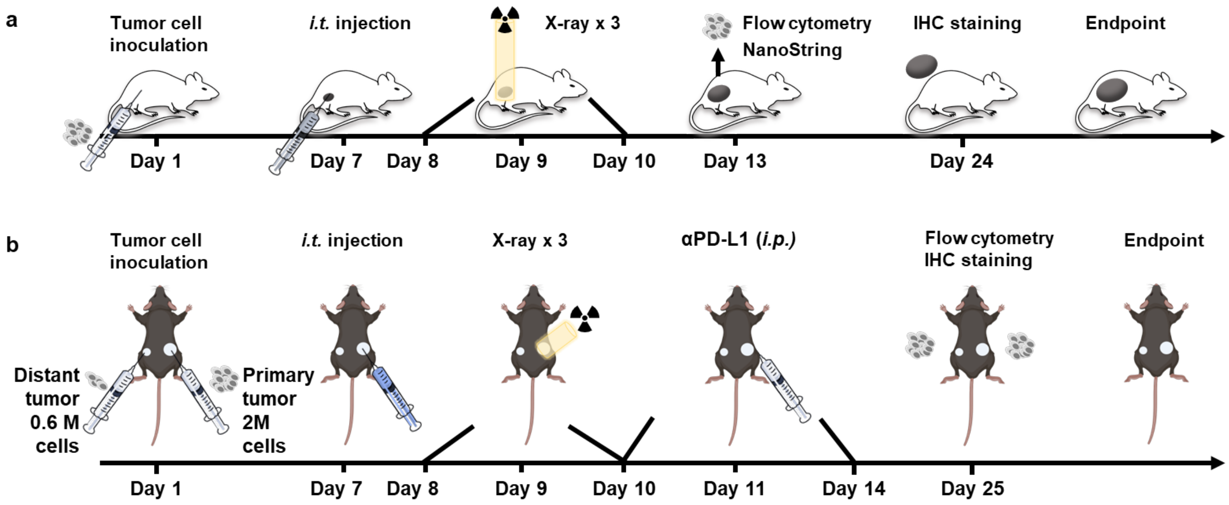

Supplementary Fig 13. Treatment schedules for tumor-bearing mice

(a) The dosing schedule and analysis schedule for single subcutaneous tumor-bearing mice. (b) The treatment schedule for bilateral tumor-bearing mice.

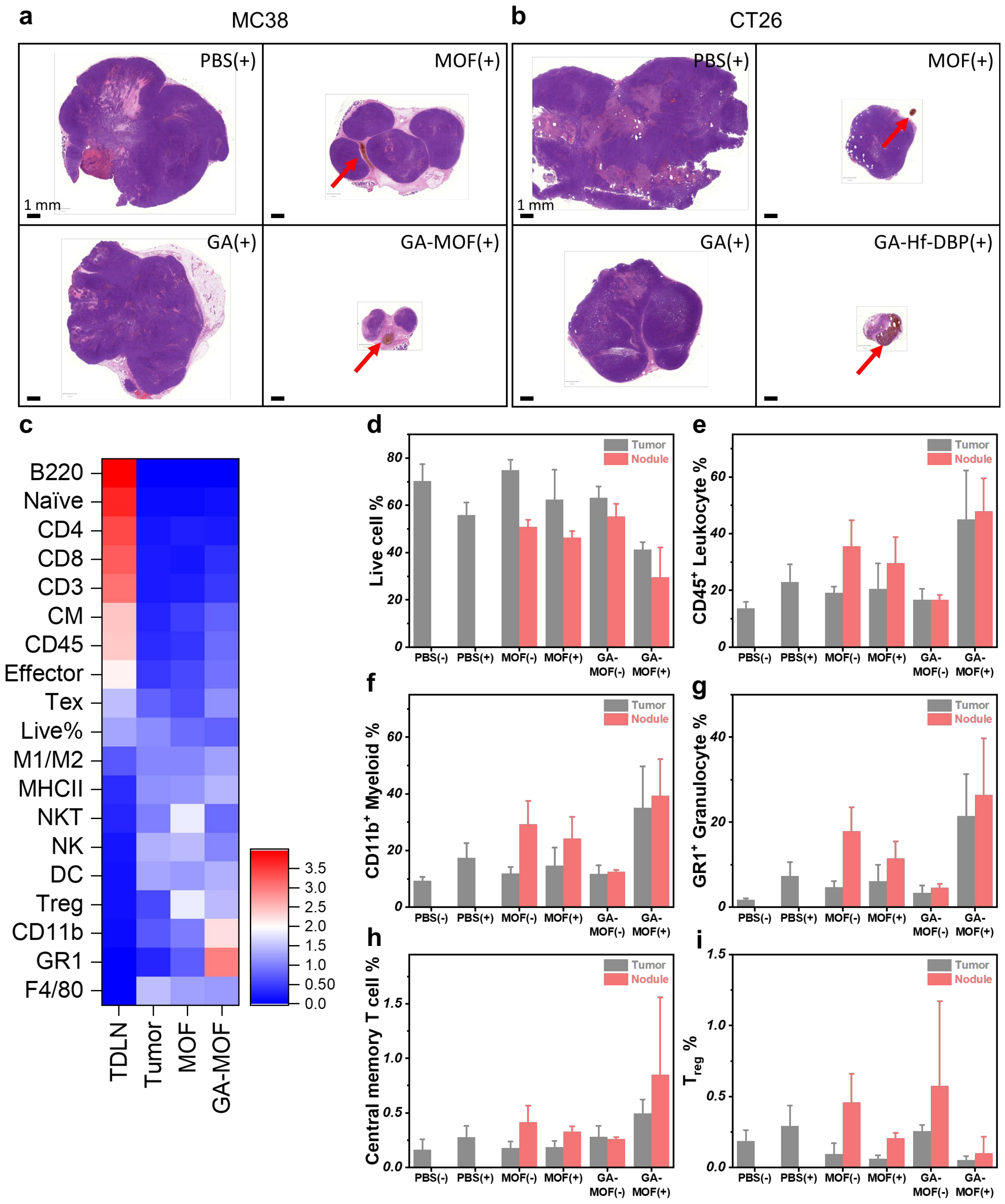

Supplementary Fig 14. Different histologies and cellular compositions between artificial leukocytoid structures (ALS) and tumors

(a-b) HE staining of whole MC38 (a) and CT26 (b) tumors with the red arrows showing the location of ALS (scale bar = 1 mm). (c-i) Heatmap (c) and detailed percentages of live cells (d), leukocytes (e), myeloid cells (f), granulocytes (g), memory T cells (h), and regulatory T cells (i) showing different immune cell populations between ALS and tumors.

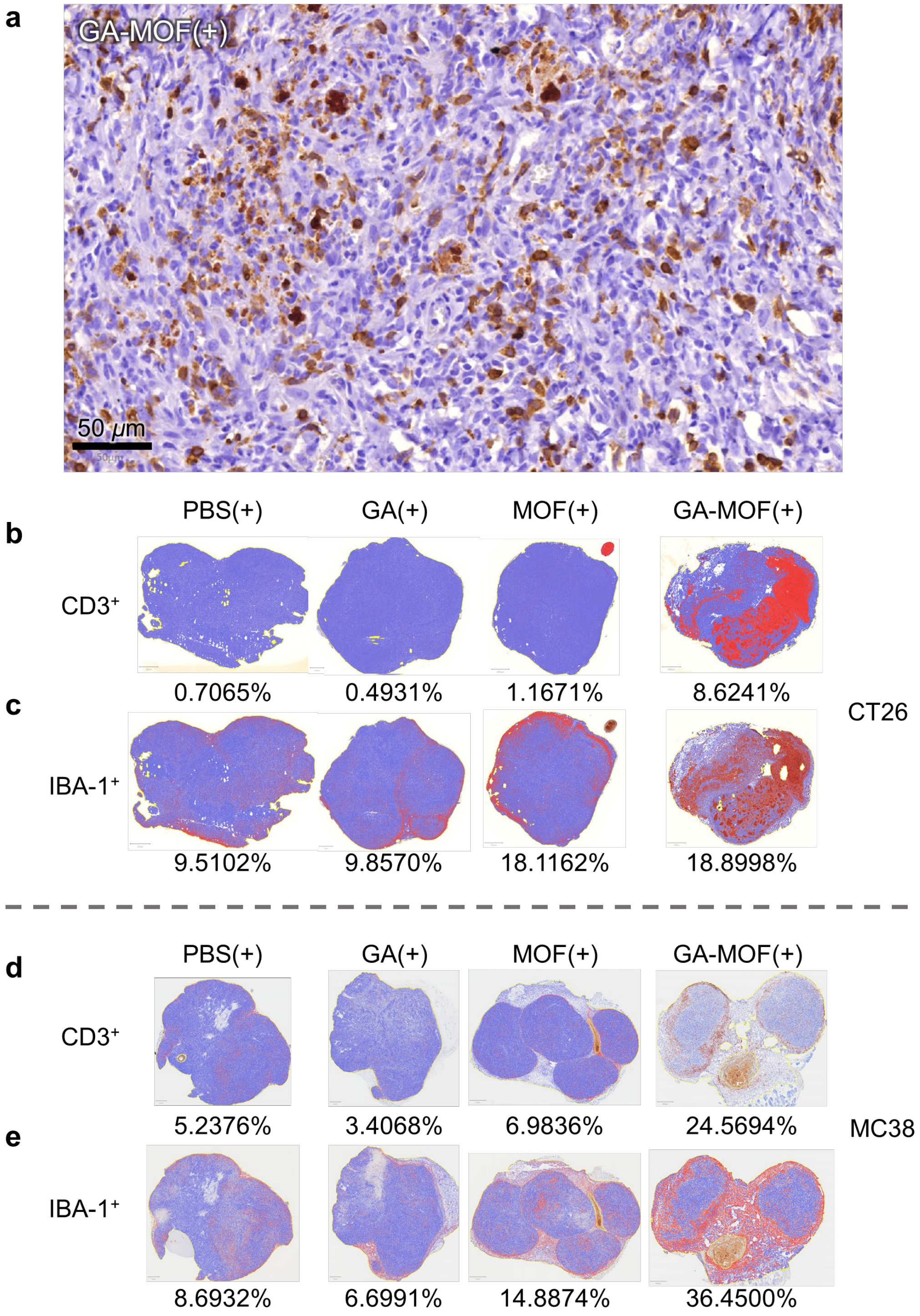

Supplementary Fig 15. Histological observation of immune cell infiltration induced by GA-MOF treatment in colon cancer models

(a) A zoomed-in view of CD3 staining in the tumor treated with GA-MOF(+) (scale bar = 20 μm). (b-c) IHC analysis of T cells (b) and pan-macrophage cells (c) in the whole CT26 tumor. (d-e) IHC analysis of T cells (d) and pan-macrophage cells (e) in the whole MC38tumor.

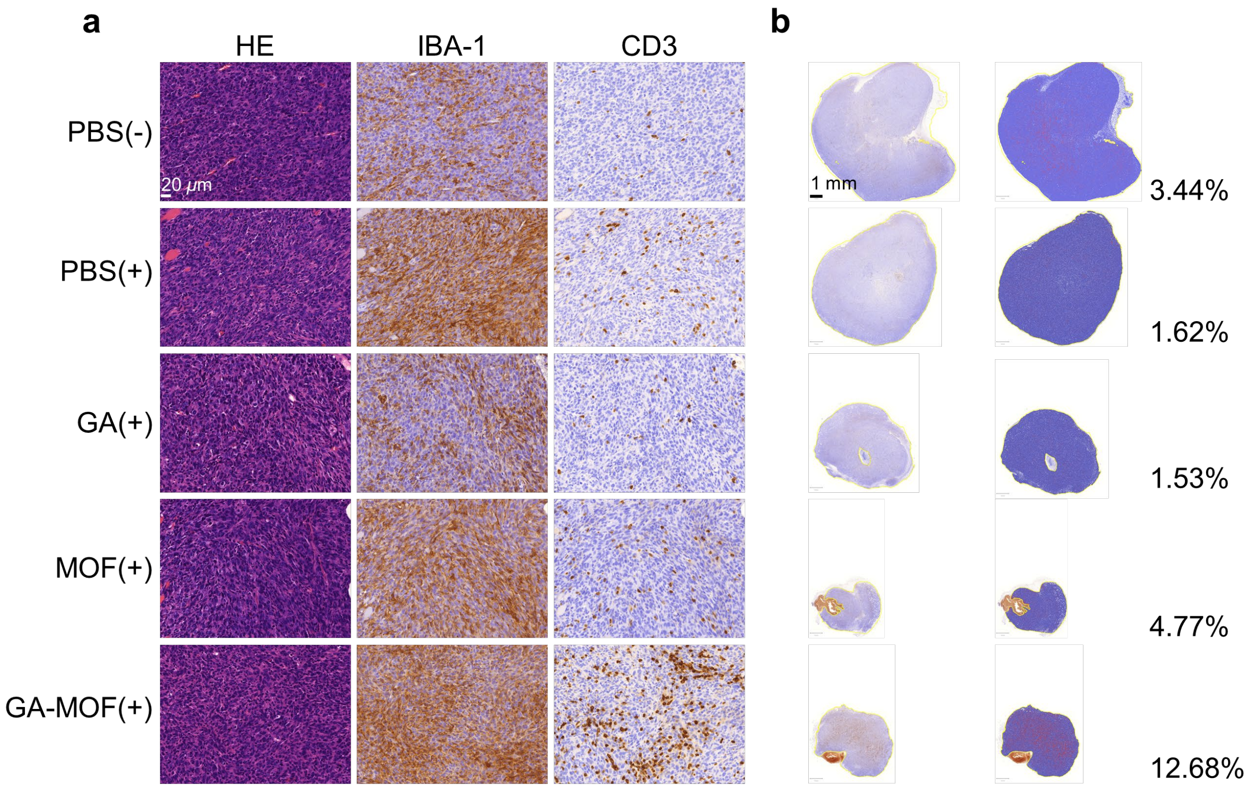

Supplementary Fig 16. Histological observation of immune cell infiltration induced by GA-MOF treatment in SCC7 head and neck cancer model

(a) A zoomed-in view of HE, IBA-1, and CD3 staining in the SCC7 tumors with different treatments (scale bar = 20 μm). (b) IHC analysis of T cells in the whole SCC7 tumor (scale bar = 1 mm).

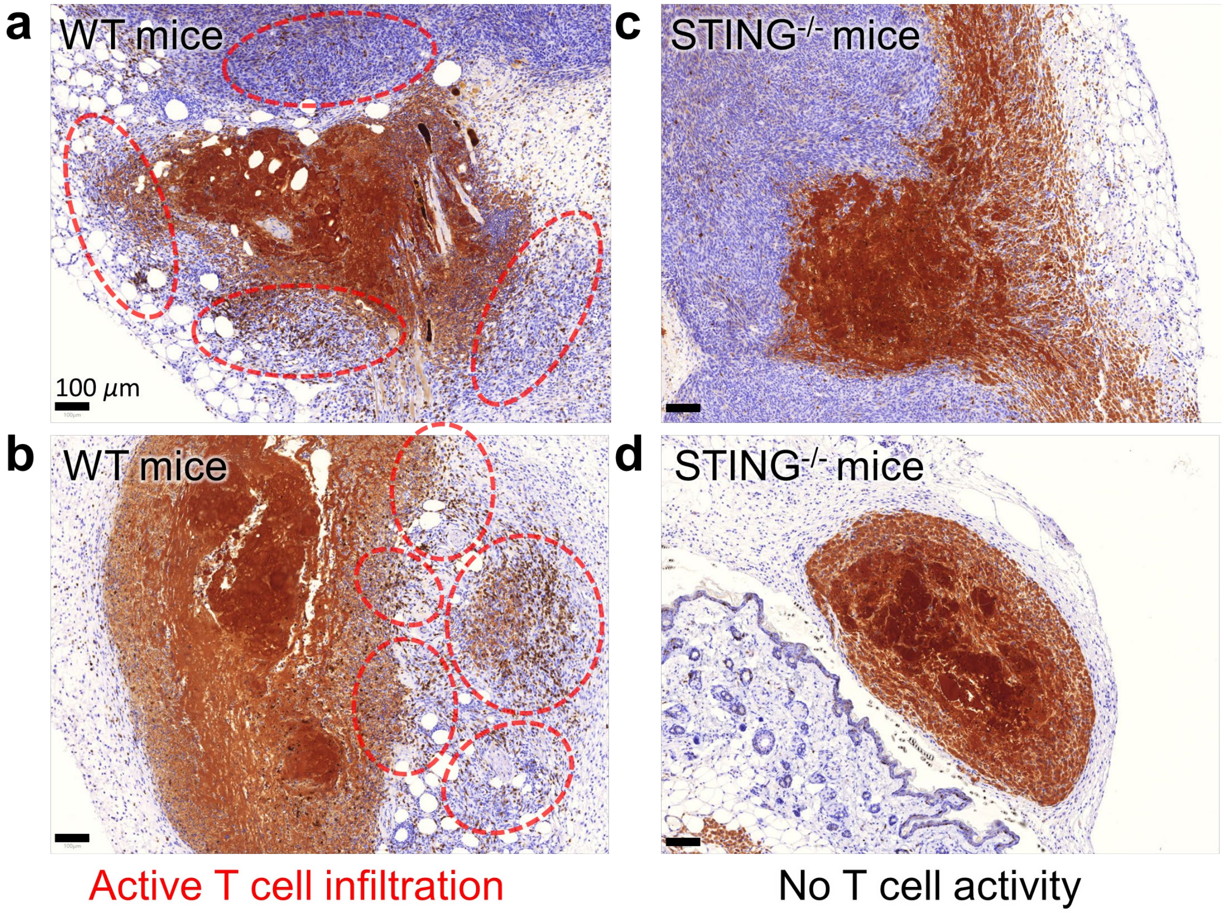

Supplementary Fig 17. Histological observation of immune infiltration near artificial leukocytoid structures (ALS) in STING$^{-/-}$ mice

(a-b) Active T cell infiltration around ALS observed in CD3 staining of MC38 tumors and subcutaneous nodule from WT B6 mice injected with GA-MOF. (b) Bare T cell infiltration around ALS in CD3 staining of MC38 tumors and subcutaneous nodules from STING-/- mice injected with GA-MOF (scale bar = 100 μm).

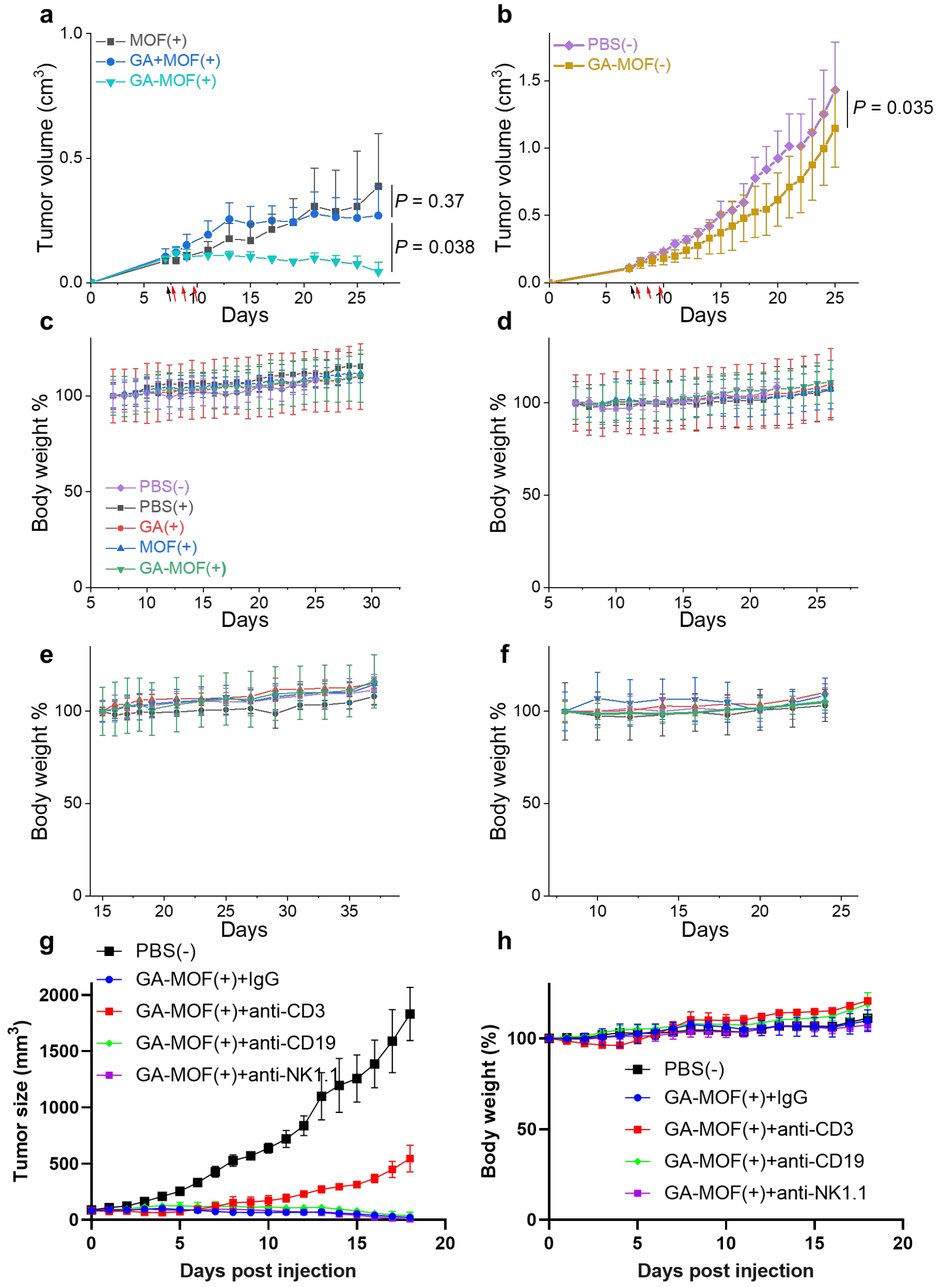

Supplementary Fig 18. Antitumor efficacy and body weights after different treatments

(a) Tumor growth curves of subcutaneous MC38-bearing C57BL/6 mice treated with MOF(+), GA-MOF(+), and GA-MOF(+) (*N*=7). "GA + MOF(+)" means GA was injected first, and MOF was injected 8 hours later. (b) Tumor growth curves of subcutaneous MC38-bearing C57BL/6 mice treated with PBS(-) and GA-MOF(-) (*N*=7). (c-f) Body weight trends of MC38 tumor-bearing C57BL/6 (c), CT26 tumor-bearing BALB/c (d), Panc02 tumor-bearing C57BL/6 (e), and SCC7 tumor-bearing C3H (f) mice in different treatment groups. (g,h) Tumor growth curves (g) and body weight trends (h) of subcutaneous MC38 tumor-bearing C57BL/6 mice treated with PBS(-), GA-MOF(+) + IgG isotype control, GA-MOF(+) + anti-CD3 antibody, GA-MOF(+) + anti-CD19 antibody, and GA-MOF(+) + anti-NK1.1 antibody (N=3).

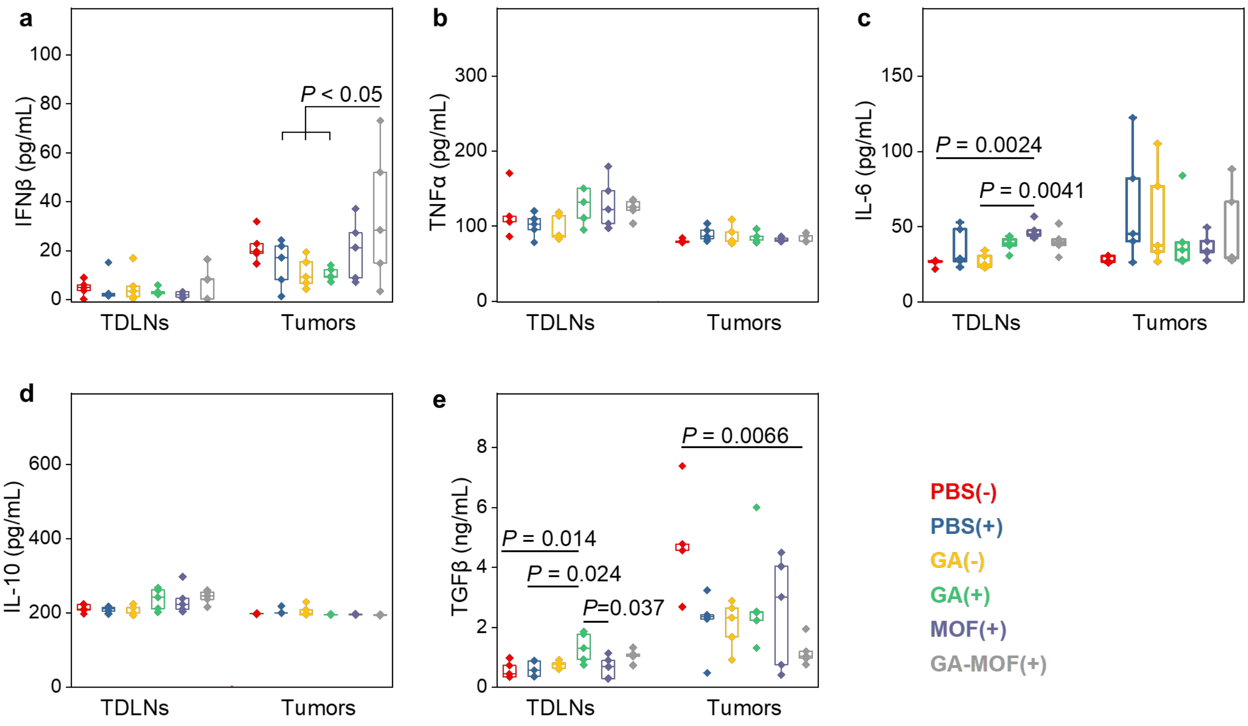

Supplementary Fig 19. Intratumoral cytokine levels after different treatments

Intratumoral concentrations of IFN-β (a), TNF-α (b), IL-6 (c), IL-10 (d), and TGFβ (e) in the excised MC38 tumors 3 days after the last RT dose (N=5).

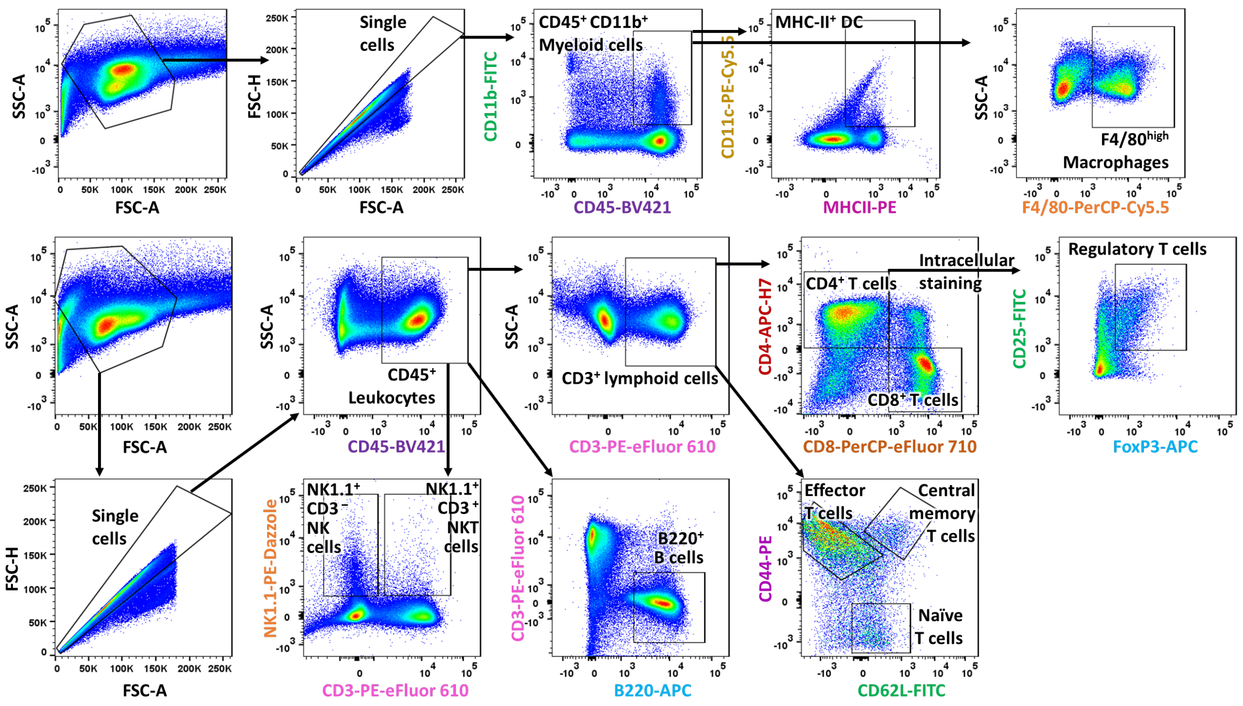

Supplementary Fig 20. Gating strategies for immune profiling of early immune responses

Gating strategies for leukocytes (CD45⁺), myeloid cells (CD45⁺ CD11b⁺), DC (CD45⁺ CD11b⁺ CD11c⁺ MHCII⁺), macrophages (CD45⁺ CD11b⁺ F4/80⁺), lymphoid cells (CD45⁺ CD3⁺), cytotoxic T cells (CD45⁺ CD3⁺ CD8⁺), helper T cells (CD45⁺ CD3⁺ CD4⁺), B cells (CD45⁺ CD3⁻ B220⁺), NK cells (CD45⁺ CD3⁻ NK1.1+), NKT cells (CD45⁺ CD3⁺ NK1.1⁺), effector T cells (CD45⁺ CD3⁺ CD44⁺ CD62L⁻), central memory T cells (CD45⁺ CD3⁺ CD44⁺ CD62L⁺), and naïve T cells (CD45⁺ CD3⁺ CD44⁻ CD62L⁺).

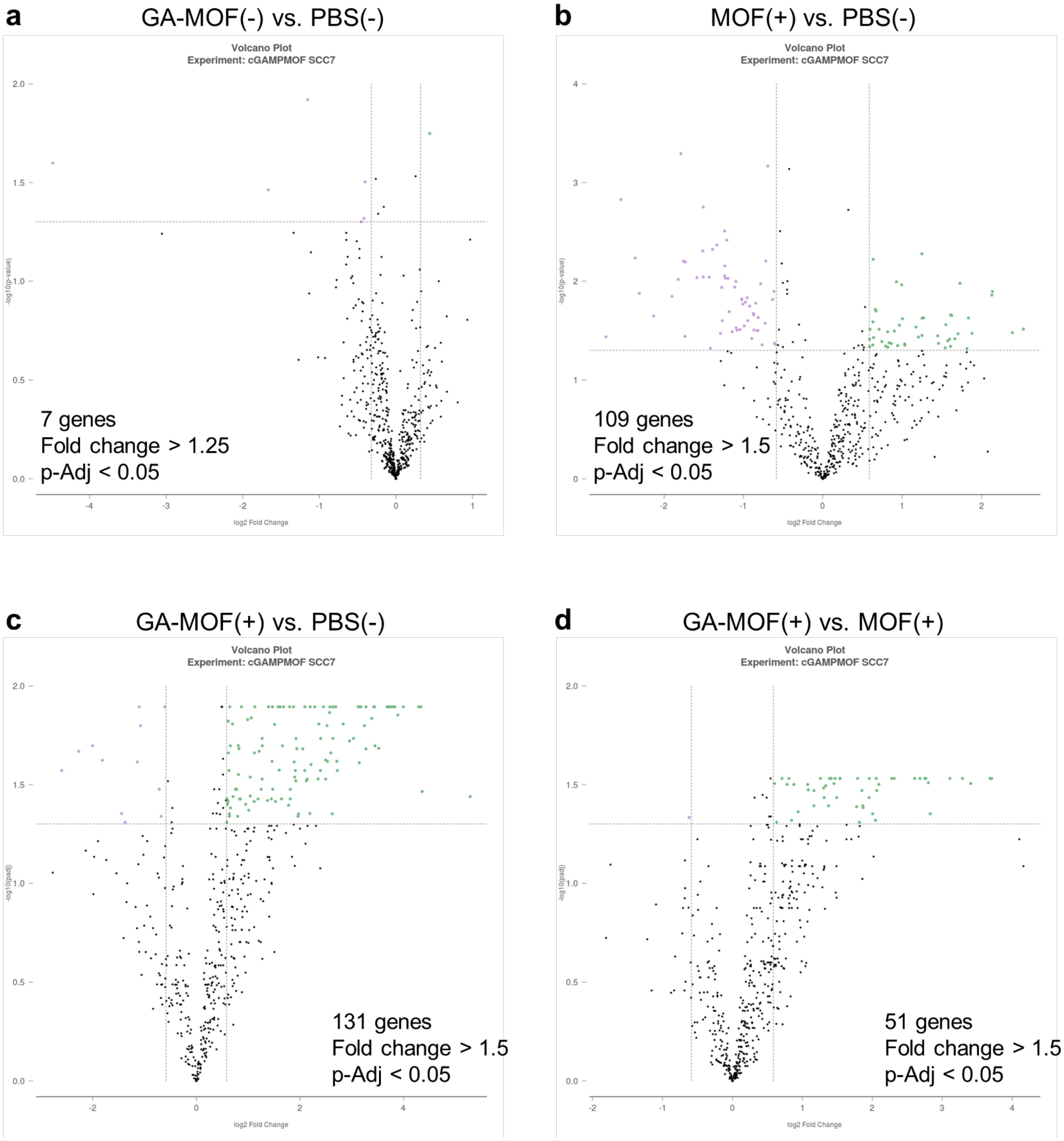

Supplementary Fig 21. Volcano plots from NanoString analysis

(a-d) Volcano plots showing genes passing the threshold for differential expressions (*N*=3) of GA-MOF(-) vs. PBS(-) (a), MOF(+) vs. PBS(-) (b), GA-MOF(+) vs. PBS(-) (c), and GA-MOF(+) vs. MOF(+) (d). Upregulation is shown in green dots and downregulation is shown in purple dots.

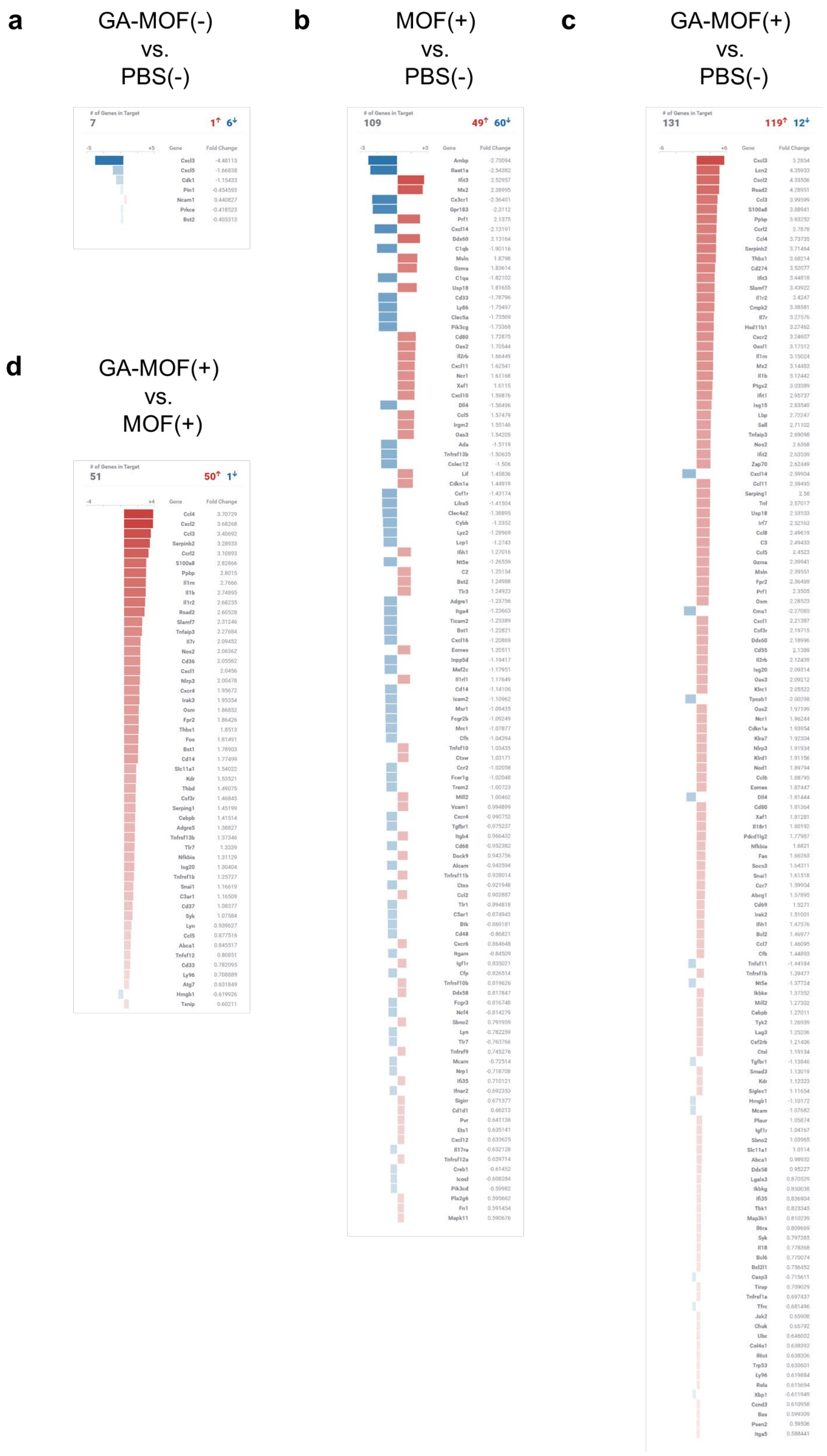

Supplementary Fig 22. Bar charts of gene lists from NanoString analysis

(a-d) Bar charts of the genes passing the threshold from Supplementary Fig. 20 showing differential expressions of GA-MOF(-) vs. PBS(-) (a), MOF(+) vs. PBS(-) (b), GA-MOF(+) vs. PBS(-) (c), and GA-MOF(+) vs. MOF(+) (d). Upregulation is shown in red and downregulation is shown in blue.

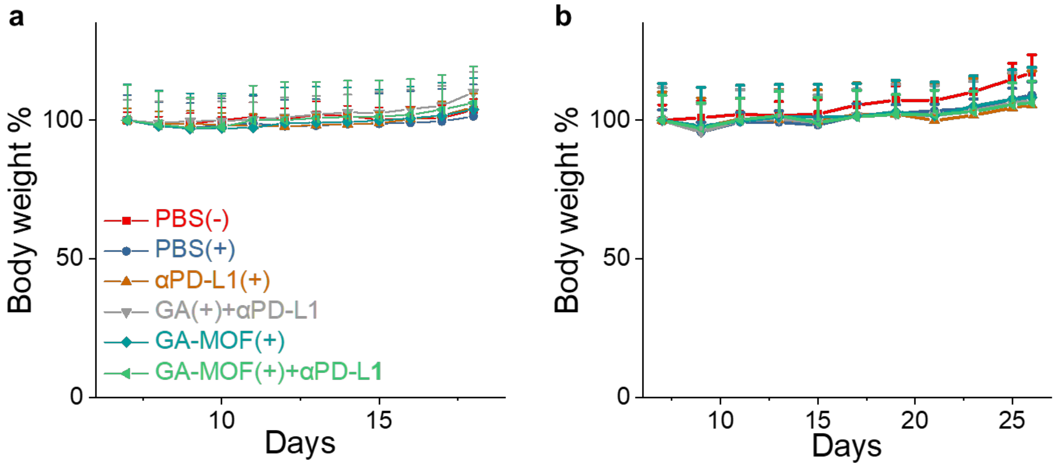

Supplementary Fig 23. Body weight curves of bilateral tumor-bearing mice

Body weight % curves of bilateral CT26 tumor-bearing BALB/c mice (a) and bilateral MC38 tumor-bearing C57BL/6 mice (b) in different treatment groups (N= 6).

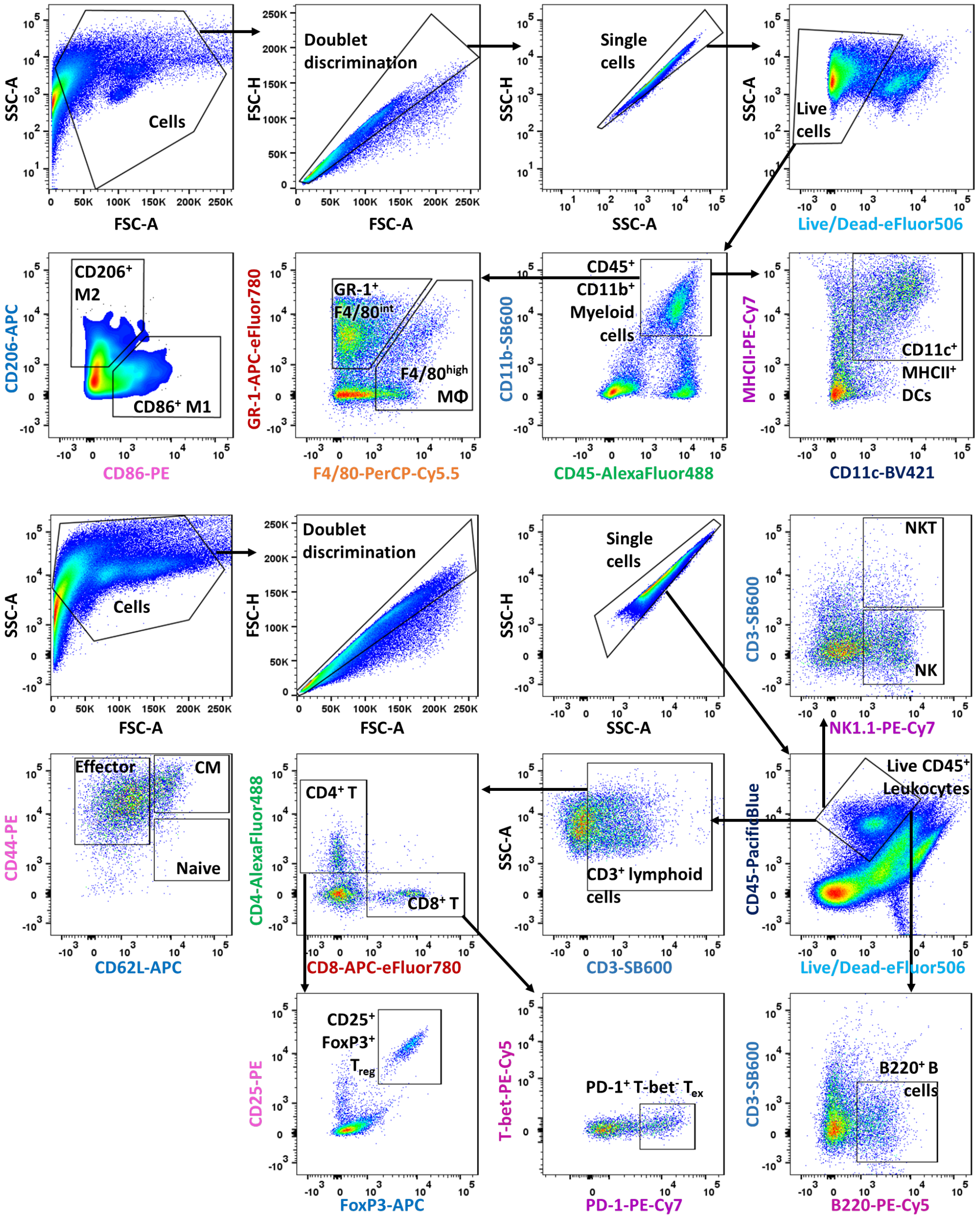

Supplementary Fig 24. Gating strategies for immune profiling of late immune responses

Gating strategies for leukocytes ($CD45^+$), myeloid cells ($CD45^+CD11b^+$), DC ($CD45^+CD11b^+CD11c^+MHCII^+$), $GR1^+$ $F4/80^{int}$ population, macrophages ($CD45^+CD11b^+GR1^{low}F4/80^+$), M1 macrophages ($CD45^+CD11b^+GR1^{low}F4/80^+CD86^+$), M2 macrophages ($CD45^+CD11b^+GR1^{low}F4/80^+CD206^+$), lymphoid cells ($CD45^+CD3^+$), cytotoxic T cells ($CD45^+CD3^+CD8^+$), helper T cells ($CD45^+CD3^+CD4^+$), B cells ($CD45^+CD3^-B220^+$), NK cells ($CD45^+CD3^-$NK1.1+), NKT cells ($CD45^+CD3^+NK1.1^+$), effector T cells ($CD45^+CD3^+CD44^+$ $CD62L^-$), central memory T cells ($CD45^+CD3^+CD44^+CD62L^+$), naïve T cells ($CD45^+CD3^+CD44^-CD62L^+$), exhausted T cells ($CD45^+CD3^+CD8^+PD\text{-}1^+T\text{-}bet^-$), and regulatory T cells ($CD45^+CD3^+CD4^+CD25^+FoxP3^+$).

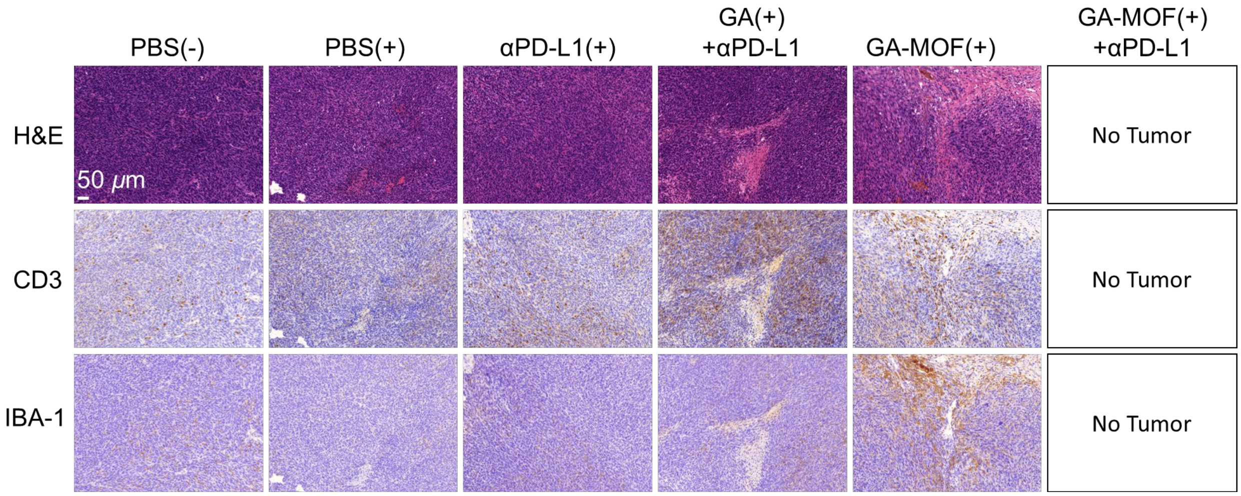

Supplementary Fig 25. Histological observation of primary MC38 tumors in the bilateral model

HE staining (top) and IHC staining of IBA-1 (top, pan-macrophage cells) and CD3 (T cells) in primary MC38 tumors in the bilateral model (scale bar = 250 μm). Because of complete eradication of MC38 tumors by GA-MOF(+)+αPD-L1, the corresponding column was left blank.

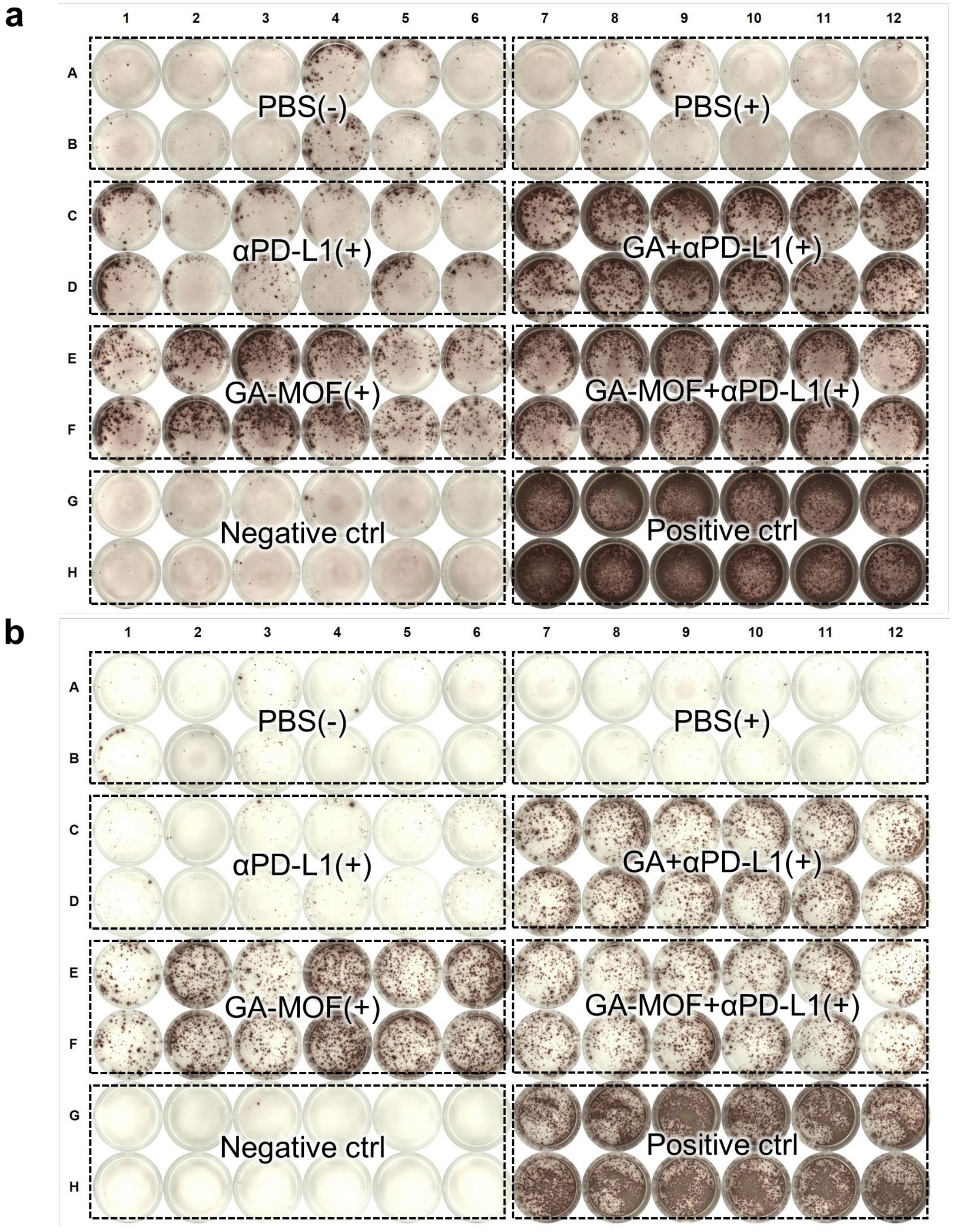

Supplementary Fig 26. Raw plate readouts for ELISpot assays

(a-b) The photos of the 96-well ELISPOT assay for CT26-bearing BALB/c mice (a) and MC38-bearing C57BL/6 mice (b) (*N*=6 with technical duplicates).

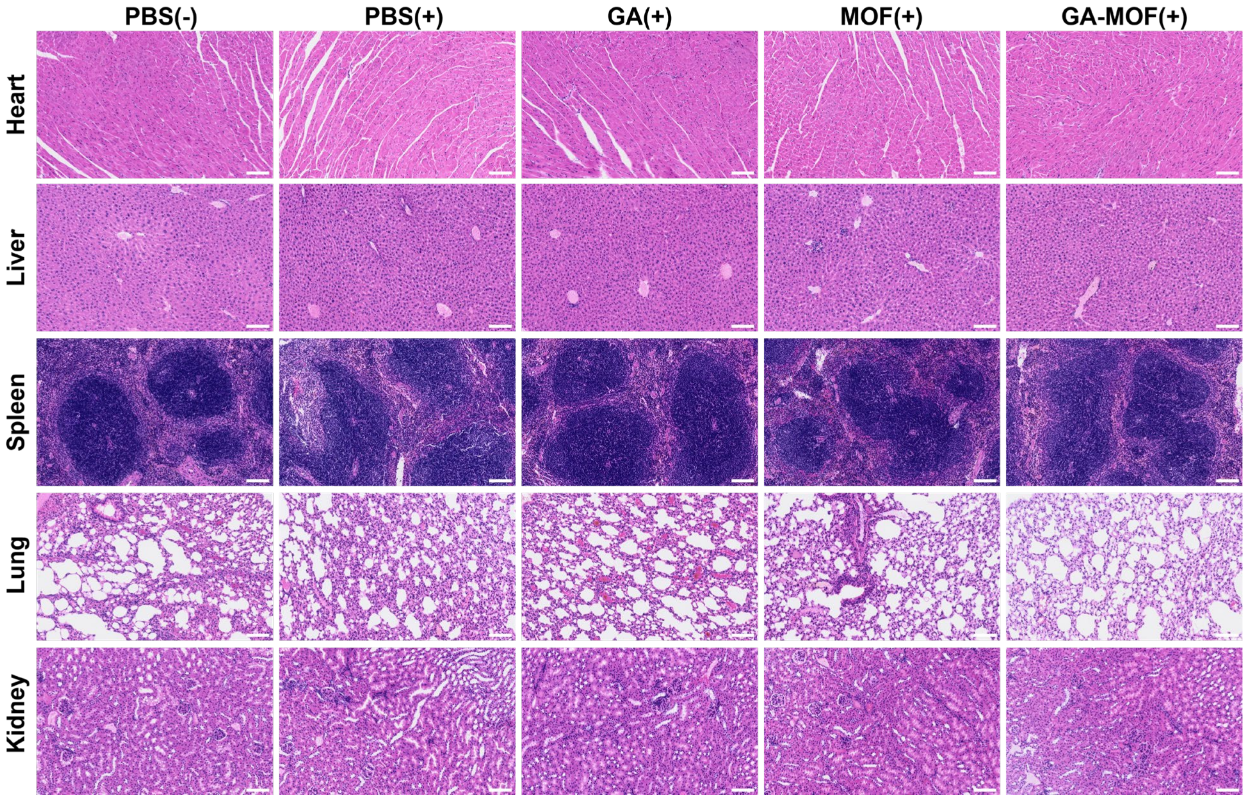

Supplementary Fig 27. Major organ histology

HE staining of major organs of MC38-bearing C57BL/6 mice after different treatments. Scale bar = 100 μm.

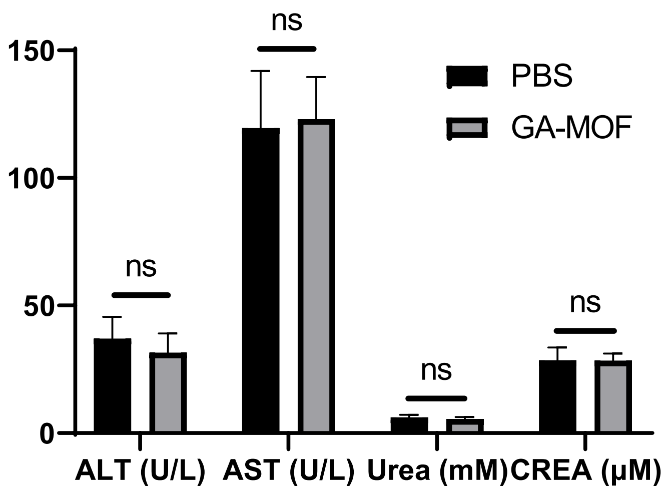

Supplementary Fig 28. Liver and kidney functions of SD rats
ALT, AST, Urea, and creatinine tests after GA-MOF treatment (*N*=10).

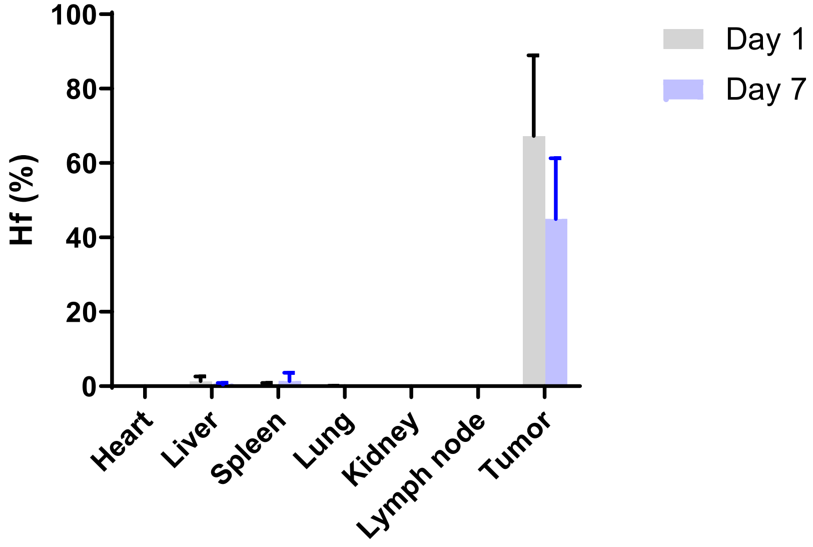

Supplementary Fig 29. Biodistribution of GA-MOF
Biodistribution of GA-MOF in MC38 tumor-bearing C57BL/6 mice one day and seven days post i.t. injection.

Supplementary Table 1. Growth rate inhibition indices (GR) and growth inhibition factors (GIF) of growth rate inhibition assays (*N*=3)

Cell Line		CT26		SCC7		MC38		Panc02	
Treatment		PBS	MOF	PBS	MOF	PBS	MOF	PBS	MOF
GR	0 Gy	1.000	1.021	1.000	1.025	1.000	1.003	1.000	0.927
	2 Gy	0.764	0.714	0.669	0.396	0.615	0.440	0.838	0.601
	4 Gy	0.493	0.402	0.204	0.191	0.323	0.214	0.525	0.411
	8 Gy	0.204	0.159	0.0326	0.0100	0.0758	0.0245	0.156	0.112
$GIF_{10\%}$		1.17		1.31		1.46		1.23	

Supplementary Table 2. TGI values of different treatment groups in four different subcutaneous murine cancer models

Treatment	$TGI_{(MC38)}$	$TGI_{(CT26)}$	$TGI_{(Panc02)}$	$TGI_{(SCC7)}$
PBS(+)	0.61	0.58	0.61	0.50
GA(+)	0.64	0.64	0.53	0.61
MOF(+)	0.71	0.83	0.71	0.75
GA-MOF(+)	0.98	0.96	0.82	0.92
GA-MOF(-)	0.09	N/A		
GA+MOF(+)	0.79			

Supplementary Table 3. Tumor growth inhibition (TGI) values of different treatment groups in the CT26 and MC38 bilateral tumor models

Cell Line	CT26		MC38	
Treatment	$TGI_{(primary)}$	$TGI_{(distant)}$	$TGI_{(primary)}$	$TGI_{(distant)}$
PBS(+)	0.56	-0.02	0.66	0.30
αPD-L1(+)	0.61	0.03	0.82	0.47
GA(+)+αPD-L1	0.75	0.41	0.88	0.64
GA-MOF(+)	0.95	-0.09	0.99	0.67
GA-MOF(+)+αPD-L1	0.98	0.66	1	0.93
